# Neuromotor variability partially explains different endurance capacities of expert pianists

**DOI:** 10.1038/s41598-023-42408-3

**Published:** 2023-09-13

**Authors:** Etienne Goubault, Craig Turner, Robin Mailly, Mickaël Begon, Fabien Dal Maso, Felipe Verdugo

**Affiliations:** 1 Laboratoire de Simulation et Modélisation du Mouvement, École de Kinésiologie et des sciences de l’activité physique, Université de Montréal, Montréal, QC Canada; 2 Sainte-Justine Hospital Research Center, Montréal, QC Canada; 3 Centre Interdisciplinaire de recherche sur le cerveau et l’apprentissage, Montréal, QC Canada; 4 Faculté de Musique, Université de Montréal, Montréal, QC Canada

**Keywords:** Risk factors, Fatigue, Electromyography - EMG, Neurophysiology

## Abstract

During fatiguing piano tasks, muscle fatigue develops differently between expert pianists. Differences in neuromotor strategies employed could explain a slower rate of fatigue development. The objective was to compare muscle activation and kinematic variabilities between *ShortDuration* (i.e., pianists with less endurance) and *LongDuration* groups. Results from 49 pianists showed that EMG activation variability of most shoulder and upper limbs muscles was greater for the *ShortDuration* group with time during two piano fatiguing tasks, namely *Digital* and *Chord* tasks. Segment acceleration variability, assessed using inertial measurement units, was also greater with time for the *ShortDuration* group at the right arm during the *Digital* task, and at the thorax and head during the *Chord* task. Finally, thorax lateroflexion variability increased with time for the *LongDuration* group (but not the *ShortDuration* group) during the *Digital* task. During the *Chord* task, wrist flexion variability was higher for the *LongDuration* group compared to the *ShortDuration* group. These results showed a direct effect of time on the pianists’ acceleration variability and EMG activation variability. In contrast, a protective effect of fatigue development could be attributed to kinematic variability. Results also suggest a higher risk of injury among pianists in the *ShortDuration* group.

## Introduction

Muscle fatigue caused by long hours of practice is critical in developing playing-related muscular disorders among pianists^1^. Muscle fatigue manifests in the upper trapezius during fast and slow music scores self-selected by pianists^2^, and in wrist and finger extensor muscles during repetitive piano tasks^3,4^. Expert pianists, with comparable years of experience, level of practice per day, maximum grip strength, laterality, age, and sex, do not show the same rate of fatigue development^3^. The pianists classified by Goubault et al.^3^ as the *ShortDuration* group showed a shorter time-to-task termination during repetitive piano tasks and a quicker myoeletric manifestation of fatigue compared to other pianists categorized as the *LongDuration* group. Previous studies showed that endurance, defined as the time until exhaustion, could be affected by different neuromotor strategies. Specifically, van Dieën et al.^5^ also discriminated two groups based on their endurance time during isometric contractions of the erector spinae, and showed a significant higher EMG activation variability in the endurance group. Oomen et al.^6^ also suggested that a lower motor variability could favor early state of fatigue and less endurance during a repetitive lifting task, and could increase the risk of development for muscular disorders. Consequently, comparing the neuromotor strategies employed by pianists of different endurance characteristics might help identify the factors slowing down the rate of fatigue development.

Laboratory studies have suggested that a greater muscle activation variability was associated with a slower rate of fatigue development^7^. Using high-density electromyography (EMG), Farina et al.^7^ showed that individuals with more heterogeneous muscle activity over time sustained static contraction longer than individuals with more uniform muscle activity distribution. Also, muscle fatigue causes adaptation; the biceps brachii, and brachialis EMG variability decreased during isokinetic, repetitive, concentric/eccentric elbow flexion^8^. Local fatigue can lead to an increased force variability in neighbor segments during isometric tasks, including maximal voluntary contraction and accurate force production before and after a 1-min maximal voluntary contraction of the index finger^9^. Also, the index finger showed a significant increase in force variability during accurate force production. Moreover, the variance of individual finger force increased for the four fingers involved in the tasks, while the total force variance showed only a modest change. This finding suggests an adaptive increase in force variability by non-fatigued segments as a strategy to slow down the rate of fatigue development, affecting accuracy during a multi-element performance. Since force fluctuations are greater as more motor units are recruited^10,11^, an association can be expected between EMG activity and its variability. This association was partially supported by Bosch et al.^12^, where the wrist extensor showed more cycle-to-cycle variability in EMG amplitude at an increased working pace. As muscle activations become more variable with fatigue, a direct impact on kinematics could be observed.

From an ergonomic viewpoint, motor variability may benefit the physiological and medical outcome of physical work as an intrinsic source of exposure variation^13^, allowing tissues to transiently recover from preceding exposures^14,15^. Although occupational studies suggest larger kinematic variability among experienced workers^16^, fatigue and recovery variability could be dependent on the measurement context^17,18^. Indeed, McDonald et al.^17^ showed that during simulated, repetitive, task-specific work, performance could be maintained throughout the post-fatigue work cycles via kinematic and muscular adaptations, such as reduced glenohumeral flexion and scapular rotation. Qin et al.^18^ observed increased kinematic variability adaptation over time in the shoulder joint to reduce the biomechanical loading on the fatigued shoulder region during a simulated, repetitive light assembly working task. On the other hand, a decreased kinematic variability over time in the more distal part of the upper limb was also observed, possibly to compensate for the increased variability caused by the shoulder joint while still maintaining task requirements^18^. These studies agree with the theory that stabilization and adaptation of voluntary movement against perturbations, like fatigue, relies on task-dependant synergies of degrees of freedom and a flexible binding^19^. This allows a stable linking of combinations of degrees of freedom that are essential to achieve the task, but releasing from tight control combinations of degrees of freedom that lead to the same task performance^20,21^. Therefore, kinematic adaptations observed to enhance endurance can happen in different body segments depending on the task performed. Kinematic variability strategies may differ between expert pianists and could explain the observed differences in time-to-task termination^3^.

This study aimed to understand the neuromotor strategies that slow down the development of fatigue in piano performance. To this end, we compared muscle activation and kinematic variabilities of expert pianists of different endurance times by assessing the effect of group (*ShortDuration* vs. *LongDuration*) and time (task *Initiation* vs. task *Termination/Midpoint* task) on the EMG and kinematic variability. We hypothesized that the *LongDuration* group would show greater EMG and kinematic variability than the *ShortDuration* group. We also hypothesized that EMG and kinematic variability would decrease with time.

## Methods

The methods described below were part of a larger study where initial results were previously published in Goubault et al.^3^. Thus, the information in this section matches the methods section of that study.

### Participants

Fourty-nine expert pianists were recruited. All participants were enrolled in undergraduate or graduate studies in piano performance or had at least a university (or equivalent) degree. Participants had to be free of any playing-related muscular disorders during the year preceding the experimentation. After receiving instructions on the protocol, participants read and signed written informed consent. The protocol was approved by the Université de Montréal’s Ethics Committee (CPER-18-086-D), and the research was performed in accordance with the Declaration of Helsinki. As described in our previous study, participants were categorized into two groups, *ShortDuration* and *LongDuration*, based on their duration on a *Digital* (repeating a right-hand 16-tone sequence) and a *Chord* (repeating a right-hand chord sequence) task^3^, as described in full details hereafter. Briefly, a k-means clustering and a silhouette validity index were used to identify the optimal number of sub-groups, i.e., two, based on the participants’ time-to-task termination, which varied across participants in the tasks described hereafter. Characteristics of participants are shown in Table 1.

**Table 1:** Characteristics of participants (mean ± SD).

	*Digital* task			*Chord* task		
	*ShortDuration*	*LongDuration*	Statistical test	*ShortDuration*	*LongDuration*	Statistical test
	N = 30	N = 19		N = 26	N = 23	
Left-handed	2	5	*X*^2^(1.49) = 2.24; *p* = 0.13	5	2	*X*^2^(1.49) = 0.41; *p* = 0.52
Sex	9♀	10♀	*X*^2^(1.49) = 2.51; *p* = 0.11	11 ♀	8 ♀	*X*^2^(1.49) = 0.29; *p* = 0.59
Time to termination (s)	209.7 ± 99.6	693.2 ± 72.7	t(47) = − 19.29; ***p* < 0.001**	257.2 ± 97.4	686.2 ± 72.8	t(47) = − 17.24; ***p* = < 0.001**
Age (y)	27.47 ± 8.80	29.84 ± 8.62	t(47) = − 1.24; *p* = 0.221	27.42 ± 5.08	29.61 ± 7.90	t(47) = − 1.16; *p* = 0.250
Mass (kg)	66.51 ± 9.98	66.45 ± 13.52	t(47) = 0.24; *p* = 0.809	65.26 ± 11.42	67.33 ± 13.24	t(47) = − 0.84; *p* = 0.399
Height (m)	1.75 ± 0.10	1.70 ± 0.08	t(46) = 1.99; *p* = 0.052	1.72 ± 0.10	1.74 ± 0.09	t(46) = − 0.47; *p* = 0.637
Experience (y)	19.23 ± 6.20	21.97 ± 8.64	t(47) = − 1.29; *p* = 0.202	19.25 ± 6.50	21.65 ± 7.97	t(47) = − 1.16; *p* = 0.252
Practice (h/day)*	3.41 ± 1.49	4.08 ± 1.18	t(47) = − 1.66; *p* = 0.102	3.26 ± 1.49	4.07 ± 1.18	t(47) = − 1.08; *p* = 0.359
Handgrip force (N)	319.1 ± 85.3	290.2 ± 75.8	t(47) = 1.20; *p* = 0.234	304.2 ± 83.3	312.8 ± 82.4	t(47) = − 0.36; *p* = 0.717
RPE at task *Term./Mid*	6.93 ± 0.52	3.53 ± 1.47	t(47) = 11.67; ***p* < 0.001**	7.27 ± 0.60	3.39 ± 1.50	t(47) = 12.13; ***p* < 0.001**
Incomplete cycles	1.9 ± 2.0	1.7 ± 2.0	t(47) = 0.32; *p* = 0.753	6.4 ± 4.5	5.9 ± 3.6	t(47) = 0.37; *p* = 0.72
Key velocity variance	7.6 ± 4.9	7.3 ± 4.6	t(782) = 0.86; *p* = 0.39	64.5 ± 141.7	50.6 ± 117.3	t(243) = 0.83; *p* = 0.41
Timing variance	1.1e−4 ± 5.8e−5	1.0e−4 ± 5.5e−5	t(47) = 0.54; *p* = 0.590	3.2e−4 ± 4.2e−4	3.5e−4 ± 4.4e−4	t(47) = − 0.28; *p* = 0.78

*In the past year. Bold values highlight significant differences between groups (α = 0.05). Group comparisons were performed for demographic data using a t-test or F-test when appropriate. *Term.* (i.e., *Termination*) is the average time instant at which the *ShortDuration* group stopped the task. *Mid.* (i.e., *Midpoint*) is the average midpoint instant at which the *LongDuration* group was analyzed, as it best matches the time instant at which the *ShortDuration* stopped the task (i.e., *Termination*).

### Instrumentation

**Figure 1:**
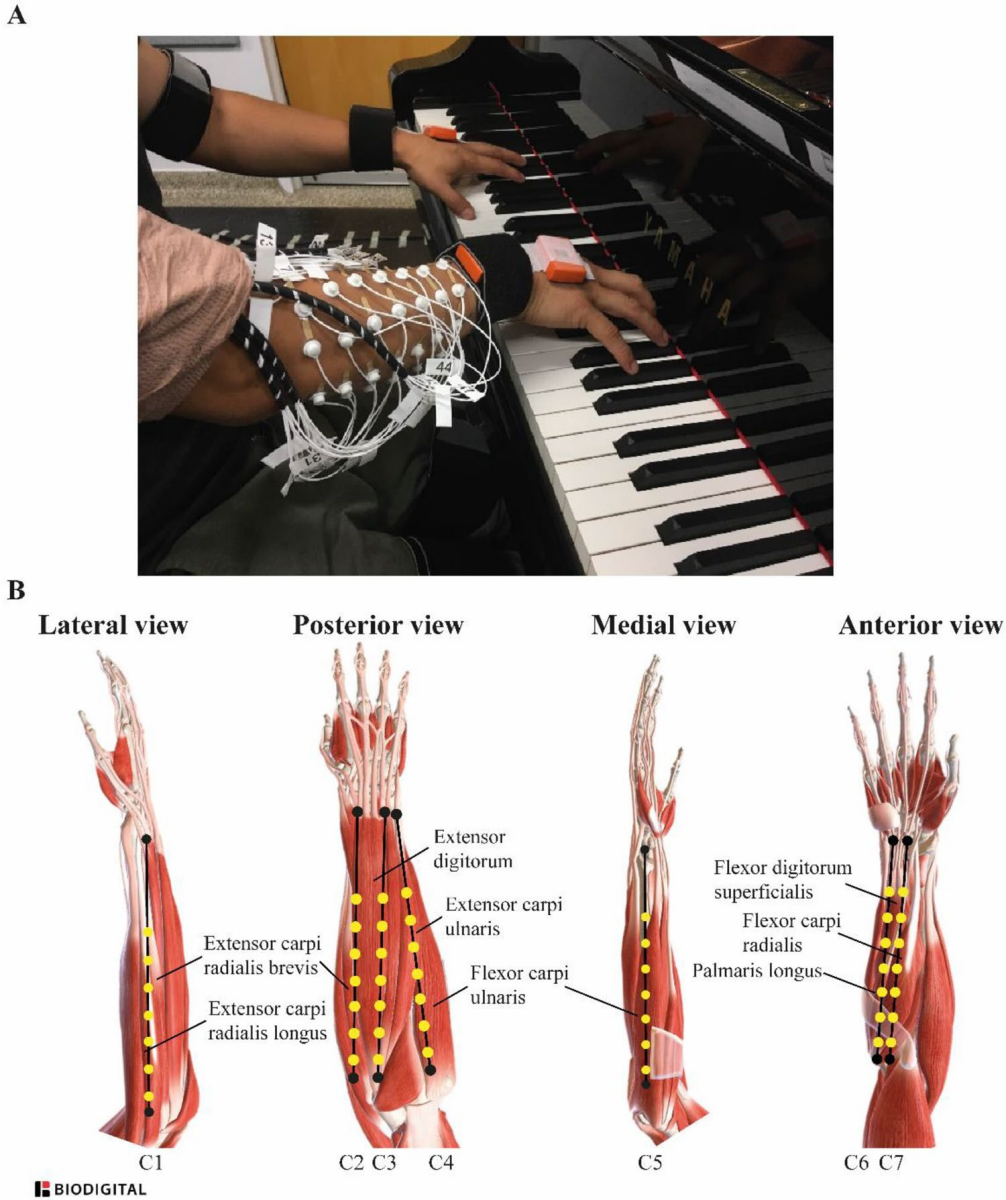
(**A**) close-up of the electrodes positioned on the forearm before medical elastic net bandage positioning, and (**B**) schematic view of the 7 × 7 array of electrodes positioned on the forearm and the underlying muscles, by Goubault et al.^3^, CC BY.

As in Goubault et al.^3^, participants were equipped with 49 monopolar EMG electrodes of 1.5 mm diameter (TMSi, EJ Oldenzaal, The Netherlands) separated by 2 cm and positioned on the right wrist flexor and extensor muscles according to a 7 × 7 array (Fig. 1). Before electrode placement, forearm skin was scrubbed with 70% isopropyl alcohol pads. A conductive gel was used to improve skin-electrode conductivity. Electrodes were attached to the skin with circular double-sided tape, and a medical elastic net bandage was used to hold the electrodes in place and minimize cable movements. Additionally, participants were equipped with two monopolar electrodes separated by 2 cm and positioned on the right biceps brachii, triceps brachii, anterior and lateral deltoids, and superior trapezius. Electrode locations were determined according to the Surface ElectroMyoGraphy for the Non-Invasive Assessment of Muscles project (SENIAM) recommendations^22^. Hair was removed with a razor when necessary, and skin was cleaned with alcohol swabs. The electrode positioning procedure was performed by a trained physiotherapist to ensure the reliability and physiological meaning of the data. It was then validated by a series of 10 submaximal voluntary contractions during which EMG signals were visually inspected in real-time on a display monitor. Briefly, the production of a burst of activation during a submaximal contraction was checked to ensure proper positioning of the electrodes with an appropriate signal-to-noise ratio. EMG signals were acquired using a 72-channel Refa amplifier (TMSi, EJ Oldenzaal, The Netherlands) at a sampling rate of 2048 Hz.

Participants were also equipped with 17 inertial measurement units (IMUs) (XSENS, Enschede, Netherlands) positioned on feet, shanks, thighs, pelvis, trunk, head, shoulders, arms, forearms, and hands, as recommended by the manufacturer. The IMUs positioning procedure was performed by the same researcher to ensure inter-participant reliability. IMU signals were acquired at a sampling rate of 60 Hz.

The participants played a grand piano (Disklavier DC7X Enspire Pro, Yamaha Canada Music Ltd, Toronto, Canada). A digital sound level meter (407,730 SLM, Extech Instruments, Nashua, USA) was placed at a 1.5 m distance from the back-left side of the piano bench to continuously monitor the sound intensity level.

### Experimental procedures

The experimental procedure was identical to Goubault et al.^3^. The music scores were sent to the participants at least five days before the experiment. Participants were asked to practice the two excerpts to play accurately and comfortably during the experiment. Before collecting data, participants’ maximal handgrip force was measured using a handgrip dynamometer (Takei Scientific Instruments, Tokyo, Japan). Participants performed two 5-s maximal voluntary contractions with the right hand, interspersed with a 1-min rest period. Verbal encouragement was provided during contractions. Participants then performed a 5-min warm-up session on the piano, followed by a sound check to familiarize themselves with the required sound intensity level. Participants were then asked to perform the *Digital* and *Chord* tasks described hereafter without interruption. The order of the two piano tasks was randomly assigned, and a 15-min rest period was provided to participants in-between tasks. Both tasks were performed fast and loud to increase muscle activation levels^23–25^ and accelerate the development of muscle fatigue^3^. To this end, the *tempo* was established using a metronome connected to an earphone placed in the participants’ left ear. A screen was positioned in front of the piano for the pianist to adjust the playing volume when necessary. The *Digital* task corresponded to a 16-tone right-hand digital sequence on the first two measures of Exercise no 7 from C.L. Hanon’s ‘The Virtuoso Pianist’ (Appendix-A1), spanning a major sixth interval in the middle register of the keyboard. Every note was a sixteenth (very short in duration) with no rests between each note. Participants were asked to play this task loudly (minimum threshold = 78 dB) and fast (quarter note = 112 beats per minute [BPM]) while maintaining an accurate sound intensity. The *Chord* task corresponded to a right-hand chord sequence based on bar 119 of Franz Liszt’s Ballade no 2 in B minor S.171 (Appendix-A1). The chord sequence was composed of three notes played five times: once in the middle register (a staccato [detached] eight note) and twice (fast repetitions) in each of the middle-high and high registers of the keyboard. The rhythm of the two chords for each fast repetition was different, with the first chord as a sixteenth note and the second chord as a staccato eight note. There was the equivalent of an eighth note rest between all the registers which, given the performance tempo, was a very small period. After the high-register chords, the rest period was slightly longer (one quarter rest and one eight rest) before the pianist had to return to the middle register for the subsequent repetition. Participants were asked to play this task loudly (minimum threshold = 84 dB [middle register] and 94 dB [high register]) and fast (quarter note = 120 BPM) while maintaining dynamic and musical intention. The rate of perceived exertion was monitored every 30 s while participants were playing via the CR-10 Borg scale^26^. Participants were stopped if they reached two consecutive scores of 7 or more at the CR-10 Borg scale or after 12 min of continuous playing^27^. Two videos extract of both *Digital* and *Chord* tasks are available in supplemental files.

### Data processing

#### Electromyography analysis

Monopolar EMG signals were converted into bipolar EMG signals by subtracting the two closest monopolar signals along the columns. EMG data were then filtered using a 10–400 Hz band-pass filter and a 60–120–180 ± 1 Hz stop-band filter to remove 60 Hz electrical contamination and its harmonics^3^. All filters were second-order zero-lag Butterworth filters. Data were then zero-aligned by subtracting the mean value. Muscle activation level was calculated using a 9 Hz low-pass filtering of the full-wave rectified EMG signals, normalized to the average of the 10 maximum amplitude values recorded during each task. Cycles, referring to each repetition of *Digital* and *Chord* excerpts, were time-normalized to handle possible variations between cycles before intra-participant variability calculation.

#### Kinematics analysis

Free-of-gravity segment accelerations (*x*, *y*, *z*) of the pelvis, thorax, neck, right scapula, shoulder, elbow, and wrist were filtered using a second-order zero-lag Butterworth band-pass filter with 0.5–15 Hz cut-off frequencies. The magnitude of segment acceleration was then calculated, and cycles were time-normalized.

Joint angles were directly extracted from XSENS MVN software (XSENS, Enschede, Netherlands), except for the pelvis and thorax, since only specific spine segments (i.e., L5, L3, T12, T8) are estimated with XSENS MVN software using interpolation of the pelvis, thorax, and head data. Therefore, pelvis and thorax angles were reconstructed using quaternion data and ‘*yxz*’ Euler angle sequence. Joints of interest were located at the pelvis (3 degrees-of-freedom [DoFs] in tilt-elevation-rotation), thorax (3 DoFs in flexion-lateroflexion-rotation), neck (3 DoFs in flexion-lateroflexion-rotation), and right scapula (3 DoFs in tilt-elevation-rotation), shoulder (3 DoFs in flexion-abduction-rotation), elbow (2 DoFs in flexion-pronation), and wrist (2 DoFs in flexion-abduction). For each DoF, the angle cycles were time-normalized.

### Measure of variability

**Figure 2:**
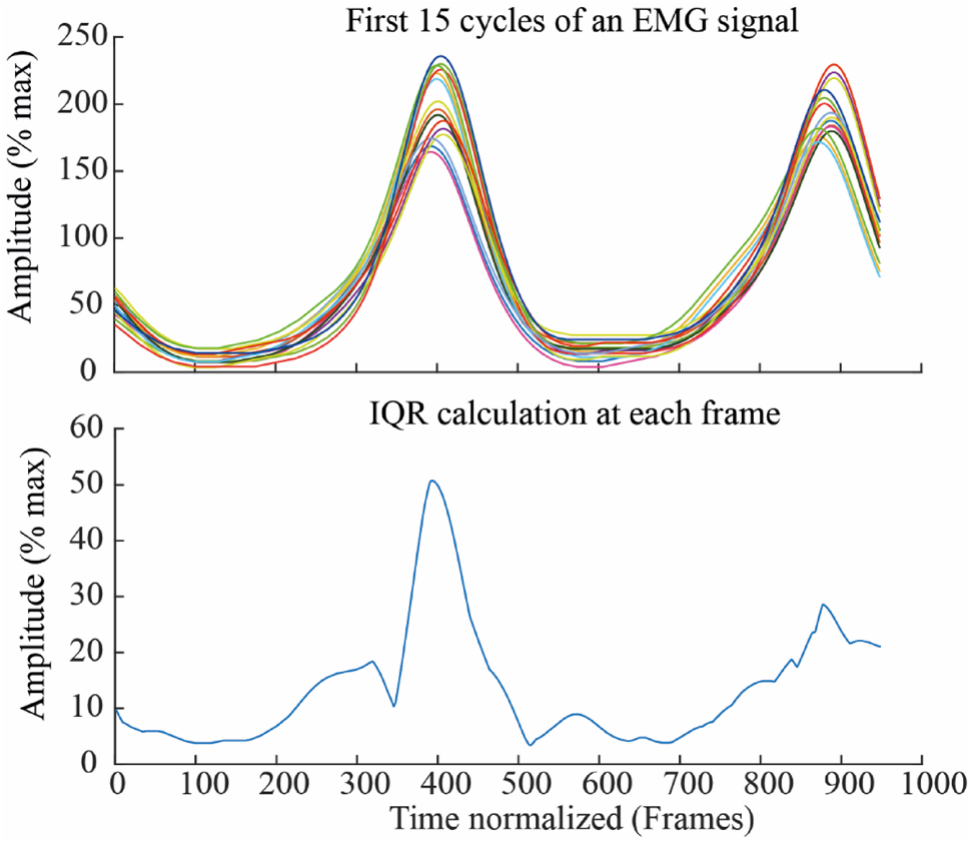
Illustration of IQR calculation at Initiation for one EMG signal of one participant. The median value of the calculated IQR curve was then used as the intra-participant variability measure.

For the *ShortDuration* group, the interquartile range (IQR) was calculated for each muscle activation, segment acceleration, and joint angle at each frame for the first and last 15 cycles (task *Initiation* and *Termination*, respectively), as illustrated on Fig. 2. For the *LongDuration* group, the interquartile range (IQR) was calculated for each muscle activation, segment acceleration, and joint angle at each frame for the first (task *Initiation*) and mid (task *Midpoint*) 15 cycles, i.e., corresponding approximately to the task *Termination* time of the *ShortDuration* group. For each participant, the median IQR at *Initiation* and *Term./Mid.* were used as the intra-participant variability measure.

### Statistical analysis

A two-way ANOVA on Group (*ShortDuration* vs. *LongDuration*) × Time (*Initiation* vs. *Term./Mid.*) with repeated measures on the Time was performed on the variability of EMG activation level, segment accelerations, and joint angles. T-test analysis was performed post-hoc as necessary. Data processing and statistical analyses were performed with Matlab R2019a (The MathWorks Inc., Natick, MA, USA).

## Results

Three and two out of fourty-nine participants were removed from analysis for the *Digital* and *Chord* tasks, respectively, because of missing data. In addition, for angle analysis of the *Digital* task, five participants were removed because of important drift observed in angle data (i.e., more than 5°).

### Muscle activation variability

**Figure 3:**
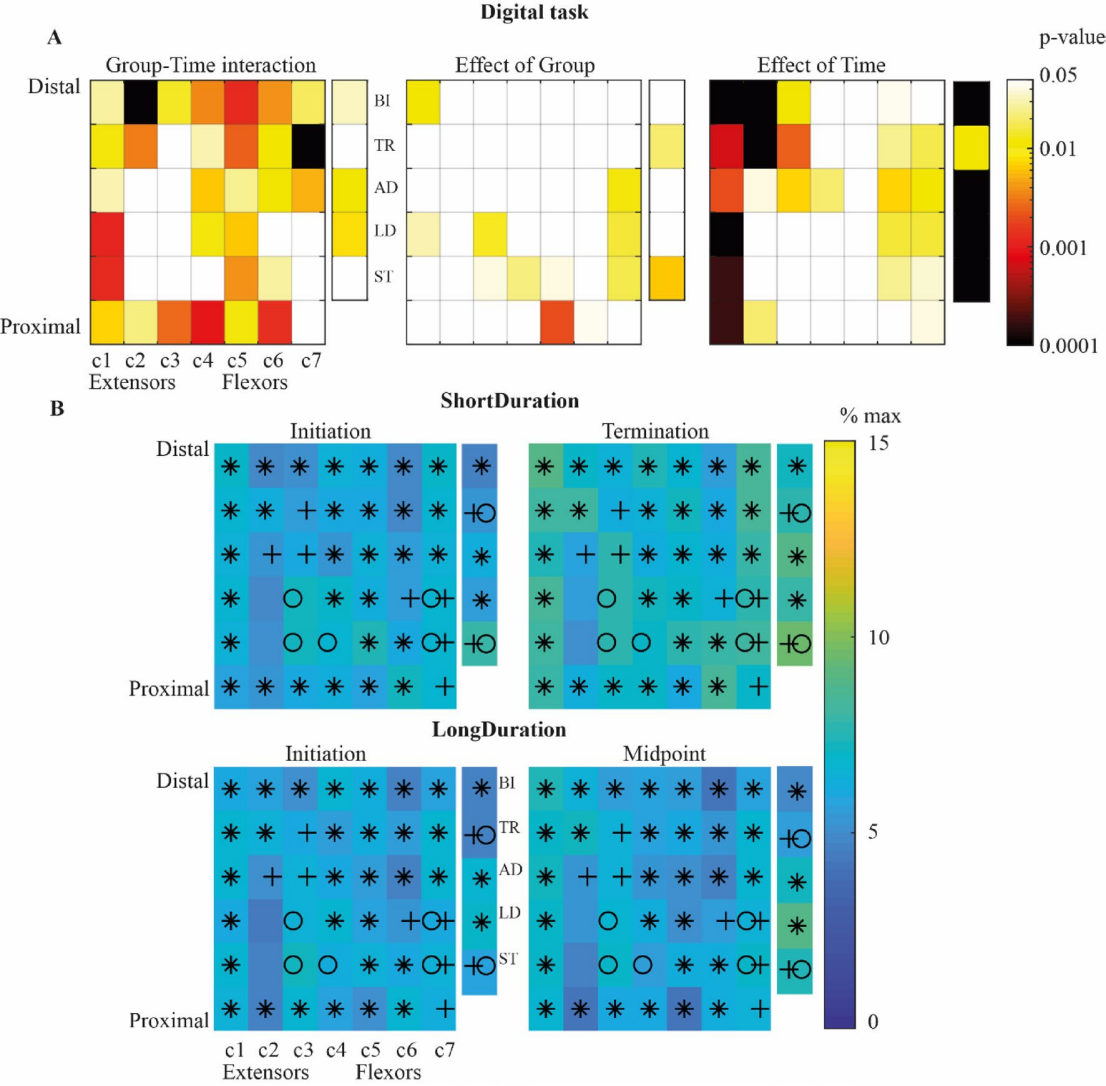
(**A**) colormap representation of ANOVA results’ *p*-values for Group-Time interaction (left), main effects of Group (middle) and Time (right) for the *Digital* task. Columns of electrodes are labeled *c1* to *c7* as illustrated in Fig. 1. *Notes*. Non-significant values, i.e., *p*-values above or equal to 0.05 are represented in white. (**B**) Median value of activation levels’ IQR at *Initiation* (left) and *Term./Mid.*, (right) for the *ShortDuration* (upper panel) and *LongDuration* (inferior panel) groups. *Notes.* *indicates significant Group-Time interaction; O indicates significant main effect of Group; + indicates significant main effect of Time; *BI* Biceps brachii; *TR* Triceps brachii; *AD* Anterior deltoid; *LD* Lateral deltoid; *ST* Superior trapezius.

*Digital task*. There was a significant Group-Time interaction for the EMG activation variability for 30 out of 42 forearm bipolar signals (Fig. 3). Paired t-test analysis showed that the EMG activation variability increased with Time for the *ShortDuration* group (*p* < 0.01), while it did not change for the *LongDuration* group (*p* > 0.05). The EMG activation variability significantly increased with Time for 6 out of 42 forearm bipolar signals. It was significantly higher for the *ShortDuration* than the *LongDuration* group for 5 out of 42 forearm bipolar signals.

In addition, there was a significant Group-Time interaction for the EMG activation variability of the biceps, anterior and lateral deltoids. Paired t-test analysis showed that the EMG activation variability increased with Time for the *ShortDuration* group (*p* < 0.001), while it did not change for anterior and lateral deltoids (*p* > 0.05) and slightly increased for the biceps (*p* = 0.01) for the *LongDuration* group. Moreover, the EMG activation variability of the triceps and superior trapezius significantly increased with Time and was significantly higher for the *ShortDuration* than the *LongDuration* group. Statistical results are shown in supplementary table S1 (Appendix-A2).

**Figure 4:**
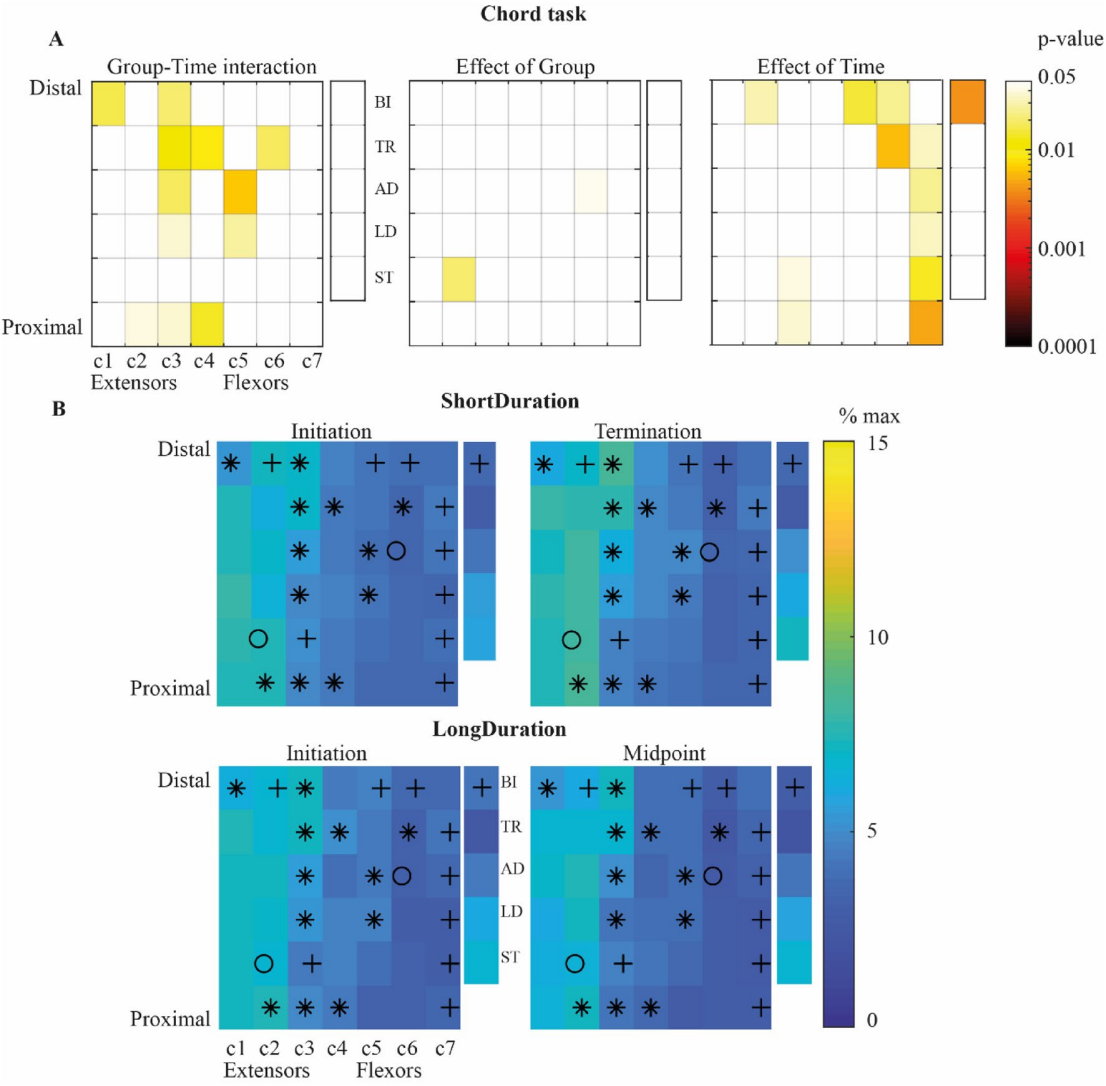
(**A**) colormap representation of ANOVA results’ *p*-values for Group-Time interaction (left), main effects of Group (middle) and Time (right) for the *Digital* task. Columns of electrodes are labeled *c1* to *c7* as illustrated in Fig. 1. *Notes*. Non-significant values, i.e., *p*-values above or equal to 0.05 are represented in white. (**B**) Median value of activation levels’ IQR at *Initiation* (left) and *Term./Mid.*, (right) for the *ShortDuration* (upper panel) and *LongDuration* (inferior panel) groups. *Notes.* *indicates significant Group-Time interaction; O indicates significant main effect of Group; + indicates significant main effect of Time; *BI* Biceps brachii; *TR* Triceps brachii; *AD* Anterior deltoid; *LD* Lateral deltoid; *ST* Superior trapezius.

*Chord task*. There was a significant Group-Time interaction for the EMG activation variability for 12 out of 42 forearm bipolar signals (Fig. 4). Paired t-test analysis showed that the EMG activation variability did not change (*p* > 0.05) and increased with Time (*p* < 0.05) for 8 and 4 bipolar signals, respectively, for the *ShortDuration* group. EMG activation variability decreased (*p* < 0.05) and was stable with Time (*p* > 0.05) for 7 and 5 bipolar signals, respectively, for the *LongDuration* group. The EMG activation variability significantly decreased with Time for 9 out of 42 forearm bipolar signals. It was significantly higher for the *ShortDuration* than the *LongDuration* group for 2 out of 42 forearm bipolar signals. In addition, the EMG activation variability significantly decreased with Time for the biceps. Statistical results are shown in supplementary table S2 (Appendix-A2).

### Segment acceleration variability

**Figure 5:**
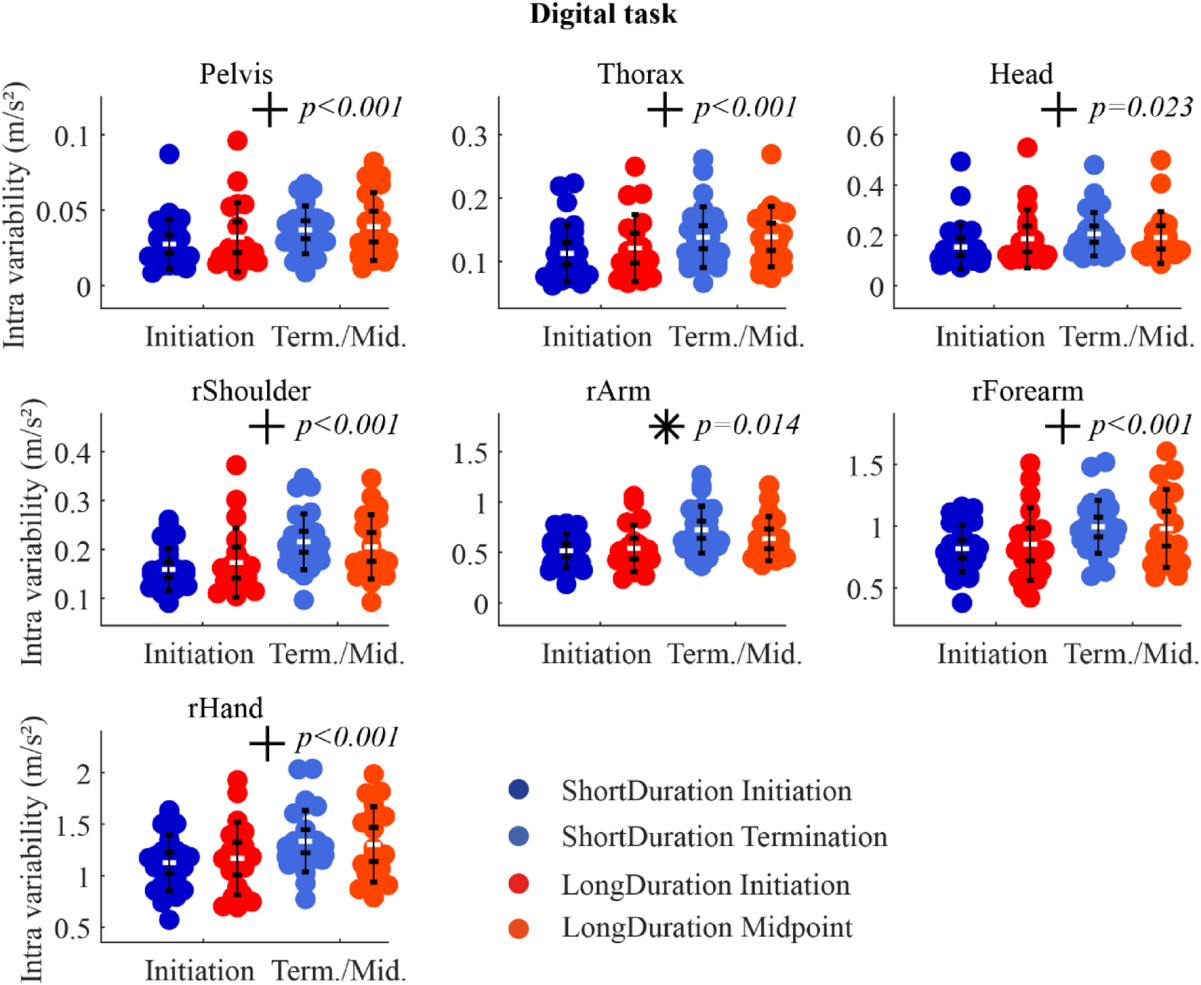
IQR of acceleration magnitude at *Initiation* and *Term./Mid.* For the *Digital* task. The white dots represent the mean, the first whisker interval represents the 95% confidence interval of the mean, and the second whisker interval represents the standard deviation of the mean. *Notes.* *indicates significant Group-Time interaction; + indicates significant main effect of Time; no main effect of Group was observed.

*Digital task* There was a significant Group-Time interaction on the variability of the right arm acceleration. Paired t-test analysis showed that the variability of the right arm acceleration increased significantly with Time for both *ShortDuration* and *LongDuration* groups (*p* < 0.001 and *p* = 0.008, respectively), with a greater increase for the *ShortDuration* group. Moreover, there was a significant increasing acceleration variability of the pelvis, thorax, head, shoulder, forearm, and hand with Time (Fig. 5). Statistical results are shown in supplementary table S3 (Appendix-A2).

**Figure 6:**
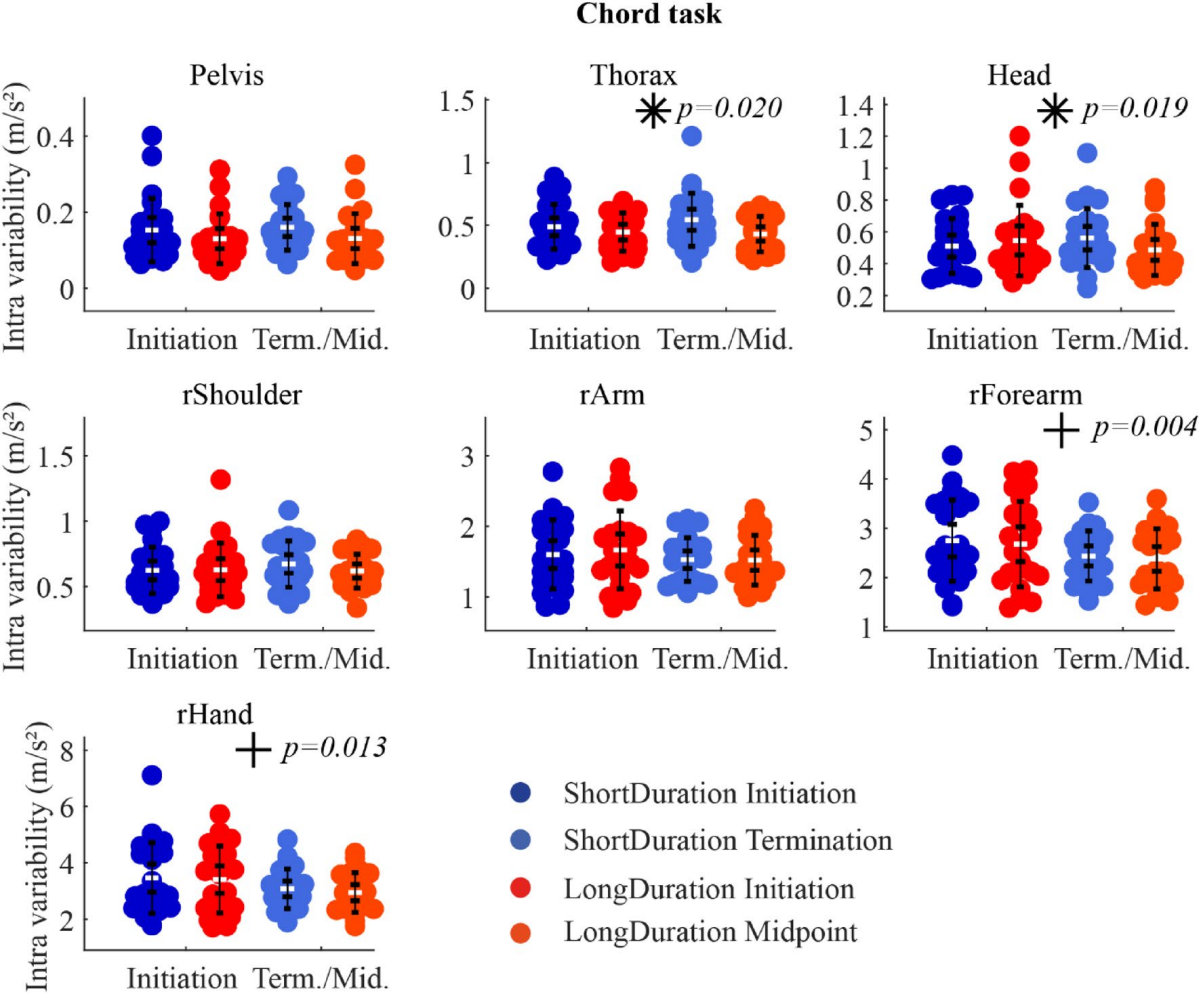
IQR of acceleration magnitude at *Initiation* and *Term./Mid.* For the *Chord* task. The white dots represent the mean, the first whisker interval represents the 95% confidence interval of the mean, and the second whisker interval represents the standard deviation of the mean. *Notes.* * indicates significant Group-Time interaction; + indicates significant main effect of Time; no main effect of Group was observed.

*Chord task* There was a significant Group-Time interaction on the variability of the thorax and head accelerations. Paired t-test analysis showed that the acceleration variability of the thorax and head increased significantly with Time for the *ShortDuration* group (*p* = 0.02 and *p* = 0.04, respectively), while it did not change for the *LongDuration* group (*p* = 0.41 and *p* = 0.15, respectively). Moreover, there was a significant decreasing acceleration variability of the right forearm and hand with Time (Fig. 6). Statistical results are shown in supplementary table S4 (Appendix-A2).

### Joint angle variability

**Figure 7:**
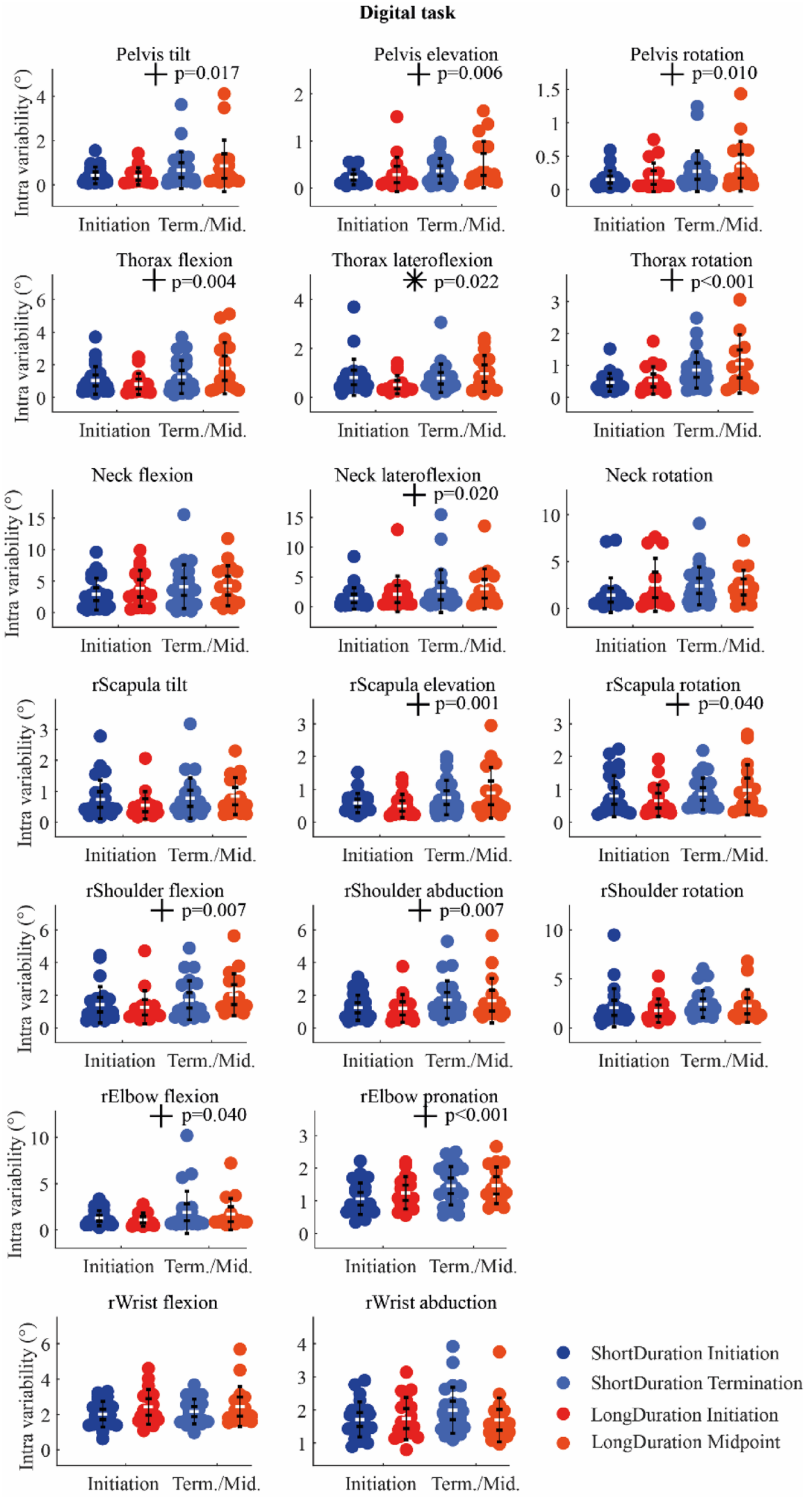
IQR of angle segment at *Initiation* and *Term./Mid.* for the *Digital* task. The white dots represent the mean, the first whisker interval represents the 95% confidence interval of the mean, and the second whisker interval represents the standard deviation of the mean. *Notes*. *indicates significant Group-Time interaction; + indicates significant main effect of Time; no main effect of Group was observed.

*Digital task* There was a significant Group-Time interaction on the variability of the thorax lateroflexion. Paired t-test analysis showed that the variability of the thorax lateroflexion increased significantly with Time for the *LongDuration* group (*p* < 0.004), while it did not change for the *ShortDuration* group (*p* = 0.81). Moreover, there was a significant effect of Time on the angle variability of the pelvis (tilt, elevation, and rotation), thorax (flexion and rotation), neck (lateroflexion), scapula (elevation and rotation), shoulder (flexion and abduction), and elbow (flexion and pronation). For all these DoFs, the angle variability increased with Time (Fig. 7). Statistical results are shown in supplementary table S5 (Appendix-A2).

**Figure 8:**
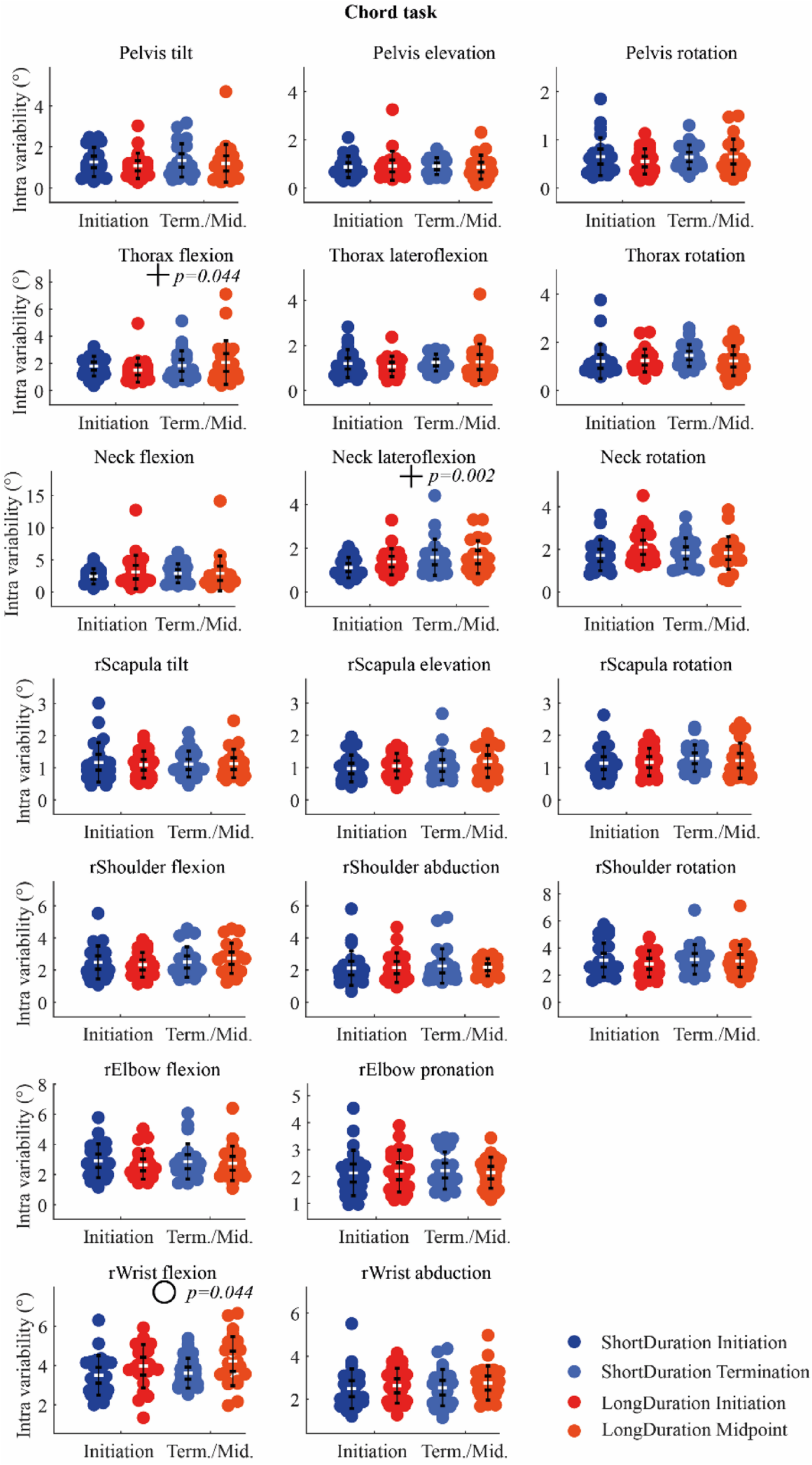
IQR of angle segment at *Initiation* and *Term./Mid.* for the *Chord* task. The white dots represent the mean, the first wisker interval represents the 95% confidence interval of the mean, and the second wisker interval represents the standard deviation of the mean. *Notes*. + indicates significant main effect of Time; o indicates significant main effect of Group; no Group-Time interaction was observed.

*Chord task* There was a significant effect of Group on the angle variability for wrist flexion, with a higher angle variability for the *LongDuration* group compared to the *ShortDuration* group. Additionally, there was a significant effect of Time on the angle variability of the thorax flexion, and neck lateroflexion; angle variability increased with Time (Fig. 8). Statistical results are shown in supplementary table S6 (Appendix-A2).

## Discussion

The objective of the present study was to understand the neuromotor strategies that slow down the development of fatigue in piano performance. Results showed that EMG activation variability increased more for the *ShortDuration* group with Time during both *Digital* and *Chord* tasks, while it did not change, or decreased for the *LongDuration* group. Additionally, EMG activation variability of the biceps, anterior and lateral deltoids increased more for the *ShortDuration* group as compared to the *LongDuration* group during the *Digital* task. In terms of kinematics, segment acceleration variability increased more with fatigue for the *ShortDuration* group at distal segments (i.e., right arm) during the *Digital* task, and at proximal segments (i.e., thorax and head) during the *Chord* task. Moreover, proximal joint angle (i.e., thorax lateroflexion) variability increased with time for the *LongDuration* group during the *Digital* task, while it did not change for the *ShortDuration* group. During the *Chord* task, distal joint angle (i.e., wrist flexion) variability was higher for the *LongDuration* group compared to the *ShortDuration* group.

### Variability and time-to-task termination

Results of the present study highlight a higher EMG activation variability with time for the *ShortDuration* group (i.e., the fatigued group), which partially contradicts previous studies suggesting either a protective effect of EMG variability against fatigue development^7^, or a decreasing EMG variability with fatigue^8^. However, in tasks that require a high level of accuracy, such as piano performance, motor variability impacting performance variables is undesirable. This could explain why the non-fatigued pianists of the *LongDuration* group maintained a low-level of EMG activation variability, whereas the fatigued pianists of the *ShortDuration* group had an increasing EMG activation variability, possibly to impede further fatigue development^5,7^. The increased EMG activation variability was accompanied by a greater increase of the right arm acceleration variability during the *Digital* task, and a greater increase of the thorax and head acceleration variability during the *Chord* task for the *ShortDuration* group, as compared to the *LongDuration* group. This possibly explains the decreased control of key-attack velocity due to fatigue for the *ShortDuration* group in Goubault et al.^3^. Interestingly, Madeleine et al.^16^ showed that experienced butchers had a larger variability than novices for several kinematic variables, but a reduced EMG variability compared to novices in simulated cutting tasks. This suggests that higher EMG variability among novices could be seen as a lack of adaptation to the work task, as seen among computer workers^28^. In the present study, the two groups had similar levels of piano experience and practice, and only differed in the time to exhaustion and myoelectric manifestation of fatigue^3^. Nonetheless, pianists from the *LongDuration* group adopted specific motor programmes, with an increased thorax lateroflexion and wrist flexion variability during the *Digital* and *Chord* task, respectively, that could have reduced the level of activity of some of the upper-limb muscles, and could possibly explain the greater time-to-task termination. An increased thorax angle variability was previously shown in repetitive fatiguing tasks^27,29,30^ suggesting a possible adaptation strategy at proximal joints used to compensate for upper-limb fatigue or fatigue-related discomfort during repetitive dynamic tasks. As pianists’ movements are individualized^31^, trunk movement could be a compensatory mechanism that potentially reduces the risk of injury. Nevertheless, these results suggest that pianists of the *ShortDuration* group could be at higher risk of injury than pianists of the *LongDuration* group, since they presented similar increasing EMG activation variability and decreasing joint angle variability as in Madeleine et al.^16^. However, future studies are needed to verify this hypothesis.

### Variability and fatigue

Results of previous studies suggest a task dependency in the evolution of EMG activation variability with fatigue. Indeed, an increasing EMG activation variability has previously been observed in arm muscles (i.e., upper trapezius, anterior deltoid, and biceps) during a repetitive fatiguing task^32,33^. In contrast, a decreasing EMG activation variability was observed with fatigue in biceps brachii and brachialis during isokinetic, concentric/eccentric elbow flexion^8^. In the present study, the time affected both *ShortDuration* and *LongDuration* groups during the *Digital* and *Chord* tasks. The EMG activation variability increased with time for some wrist and finger flexor and extensor muscles and for the triceps and superior trapezius during the *Digital* tasks. During the *Chord* task, a decreasing EMG activation variability was observed with time for some wrist and finger flexor and extensor muscles as well as for the biceps. The notion of task dependency is reinforced with kinematics adaptations, with an increasing acceleration variability with time for most segments during the *Digital* task, and a decreasing acceleration variability with time for the right forearm and hand during the *Chord* task. It is also reportable that acceleration variability of the thorax, for instance, was qualitatively four times higher during the *Chord* task compared to the *Digital* task, suggesting that pianists used more their trunk motion during the *Chord* task to produce keystrokes. To produce keystrokes, expert pianists may use multi-joint upper-limb movements^34,35^. These multi-joint movements can also involve trunk motion, which can contribute to creating hand and finger keystroke velocities^36^, to facilitating and initiating upper-limb movements^37^ as well as to increasing upper-limb joint angle variability^38^. The same observation can be made for other segments, i.e., acceleration variability was higher at the upper-limb(s) and the head during the *Chord* task compared to the *Digital* task. The different movement characteristics of both tasks^3^ could explain the differences observed in the evolution of segment acceleration variability. The *Chord* task (1) requires movements of larger amplitude than the *Digital* task, and (2) is composed of sections formed by different fast rhythms with a short rest between each section (demanding therefore the production of higher bursts of keystroke force in short time periods). Therefore, during the *Chord* task, fatigue could be at the origin of the decreasing acceleration variability of distal segments because of less movement amplitude or a negative impact on the musical intention with the development of fatigue. On the contrary, the *Digital* task (1) requires movements of less amplitude with notes very closed to each other on the keyboard, and (2) is only composed of continuous fast (sixteenth) notes without rest between each note. This means that for keeping a high level of performance (i.e., no error and the right sound intensity and timing between notes as assessed in our previous study^3^), the room for maneuver of pianists in terms of acceleration variability is very low. Therefore, an increase of acceleration variability of the segments could be interpreted as a partial “loss of control” due to fatigue in that particular case, one that probably had a direct incidence on the variance of key velocity observed in our previous study^3^. Overall, even if the acceleration variability evolved in either direction with fatigue during both *Digital* and *Chord* tasks, it may be interpreted as a consequence of fatigue development instead of a strategy to impede further fatigue progress.

### Music and variability

High inter-trial repeatability of EMG measurements was observed in string players (violinists) with less than 10% deviation of the mean muscular activities from their averages over the repetitions^39–41^. Another study showed low variability in maximum mouthpiece forces and kinematics among trumpet players^42^. The highly repeatable muscle activation and kinematic patterns in musicians, reflected in the present study by marginal variability of expert pianists muscle activation and movement patterns, could be explained by the years of practice required to become an expert^40,43^. Ericsson et al.^44^ estimated that expert musicians spent over 10,000 h of musical practice by the age of 21, leading to reorganization in certain sensory and motor systems and their interface^45^. The present study highlights that expert pianists can reproduce muscle activity and movement patterns and partially use variability with the development of fatigue when performing repetitive fatiguing *Digital* and *Chord* tasks.

### Limitations

Some limitations have been taken into consideration when interpreting the results. First, the maximal voluntary contraction was not performed for all muscles. The EMG normalization was therefore determined using the average of the 10 maximum amplitude values recorded during each task. However, this should have a limited impact on the intra-participant EMG activation variability results as each bipolar signal is compared to itself within task *Initiation* and *Term./Mid.* Then, angular joints measured using IMUs should be interpreted with caution since fusion algorithms used to estimate sensor orientations can be subject to error accumulation over time caused by environmental magnetic perturbations and drift^46–48^. Nonetheless, drift should have a limited impact on the interpretation of the results since joint angle variability was calculated over 15 cycles, representing approximately 30-s of data recording.

### Conclusion

Overall, this study highlights a direct effect of time on the EMG activation and segment acceleration variability of pianists, whereas a higher joint angle variability was observed in pianists having greater time-to-task termination. This suggests a direct effect of fatigue on EMG activation and segment acceleration variability, while a protective effect of fatigue development could be attributed to joint angle variability. Also, expert pianists with lower time-to-task termination during standard repeated *Digital* and *Chord* tasks exhibited an increased EMG activation variability, a greater increased acceleration variability and a lower wrist and trunk angle variability with time as opposed to expert pianists having higher time-to-task termination. This highlights different neuromotor strategies between pianists of different endurance characteristics, that could have an impact on the prevalence of injury. Future research should verify this hypothesis by assessing the EMG activation and kinematic variability effects from a training intervention aimed at increasing pianists’ endurance.

### Supplementary Information

Supplementary Information.

## Data Availability

The datasets used and/or analysed during the current study available from the corresponding author on reasonable request.

## Abbreviations

EMG: Electromyography
IMU: Inertial measurement units
IQR: Inter-quartile range
