# Supplementary material for: Neuromotor variability partially explains different endurance capacities of expert pianists
Source: Sci Rep. 2023 Sep 13;13:15163. doi: 10.1038/s41598-023-42408-3 (PMC10499816; doi:10.1038/s41598-023-42408-3)
Supplement: Supplementary file 1 — Supplementary Information. [file 41598_2023_42408_MOESM1_ESM.docx]

**Title: Neuromotor variability partially explains different endurance capacities of expert pianists**

**Authors:** Etienne Goubault^a^*, Craig Turner^a^, Robin Mailly^a^, Mickaël Begon^a,b^, Fabien Dal Maso^a,c^, Felipe Verdugo^a,d^

^a^Laboratoire de Simulation et Modélisation du Mouvement, École de Kinésiologie et des sciences de l’activité physique, Université de Montréal, Montréal, Québec, Canada (affiliation where the research was conducted)

^b^Sainte-Justine Hospital Research Center, Montréal, Québec, Canada

^c^Centre interdisciplinaire de recherche sur le cerveau et l’apprentissage, Montréal, Québec, Canada

^d^Faculté de musique, Université de Montréal, Montréal, Québec, Canada

***Corresponding author:** [etienne.goubault.de.brugiere@umontreal.ca](mailto:etienne.goubault.de.brugiere@umontreal.ca)

# Appendices

## A1. Experimental instruction for both Digital and Chord excerpts

***Digital task: the first two measures of the exercise no.7 of ‘The Virtuoso Pianist’ (C.L. Hanon)***


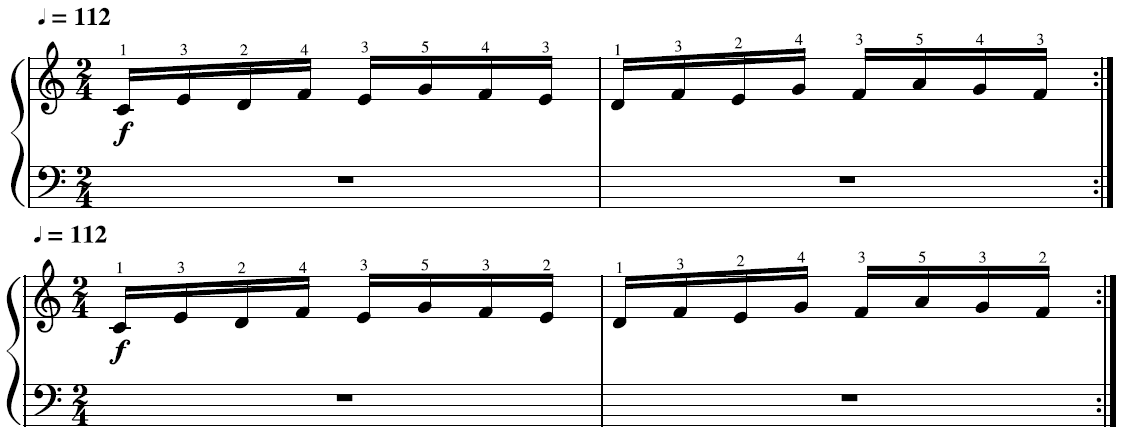


Instructions: *i)* use your right hand only (left hand resting on your left thigh); *ii)* As this excerpt can be performed using different fingering options, participants were asked to choose one fingering between two given options to facilitate standardization of experimental procedures and to allow participant to play comfortably. The chosen fingering options differed at two specific parts of the excerpt and concerned in both cases the annular, the middle, and the index fingers.

***Chord task: the 119th measure of the Ballade no.2 in B minor S.171 (F. Liszt)***

Instructions: *i)* play the bar framed by a rectangle only; *ii)* do not play the left-hand notes crossed by a X; *iii)* hold the sustain pedal from the first beat and release it at the beginning of the fourth beat for each iteration; *iv)* hold the left-hand notes until the third beat (as if they were dotted half notes). A unique and standard fingering option was given to perform this excerpt.

## A2. Statistical results tables

Table S1: Two-way ANOVA results for EMG activation variability of the Digital task.

|  |  | SumSq | DF | MeanSq | F | pValue | Ƞ² |
| --- | --- | --- | --- | --- | --- | --- | --- |
| Interaction | ST | 6,14 | 1,00 | 6,14 | 1,85 | 0,181 | 0,028 |
|  | LD | 24,95 | 1,00 | 24,95 | 7,80 | **0,008** | *0,109* |
|  | AD | 20,96 | 1,00 | 20,96 | 7,07 | **0,011** | *0,090* |
|  | TR | 8,22 | 1,00 | 8,22 | 1,90 | 0,175 | 0,036 |
|  | BI | 7,64 | 1,00 | 7,64 | 4,62 | **0,037** | *0,068* |
|  | s1 | 8,16 | 1,00 | 8,16 | 5,09 | **0,029** | *0,068* |
|  | s2 | 8,65 | 1,00 | 8,65 | 6,72 | **0,013** | *0,105* |
|  | s3 | 6,45 | 1,00 | 6,45 | 4,90 | **0,032** | *0,082* |
|  | s4 | 15,09 | 1,00 | 15,09 | 12,16 | **0,001** | **0,151** |
|  | s5 | 15,37 | 1,00 | 15,37 | 11,91 | **0,001** | **0,164** |
|  | s6 | 13,04 | 1,00 | 13,04 | 7,95 | **0,007** | *0,115* |
|  | s7 | 20,34 | 1,00 | 20,34 | 18,61 | **0,000** | **0,202** |
|  | s8 | 9,48 | 1,00 | 9,48 | 9,55 | **0,003** | *0,107* |
|  | s9 | 1,01 | 1,00 | 1,01 | 1,01 | 0,320 | 0,020 |
|  | s10 | 1,97 | 1,00 | 1,97 | 2,43 | 0,127 | 0,050 |
|  | s11 | 3,23 | 1,00 | 3,23 | 3,36 | 0,074 | *0,070* |
|  | s12 | 3,91 | 1,00 | 3,91 | 5,43 | **0,024** | *0,099* |
|  | s13 | 7,39 | 1,00 | 7,39 | 6,48 | **0,015** | *0,112* |
|  | s14 | 1,31 | 1,00 | 1,31 | 1,21 | 0,277 | 0,022 |
|  | s15 | 1,78 | 1,00 | 1,78 | 1,72 | 0,196 | 0,032 |
|  | s16 | 1,37 | 1,00 | 1,37 | 1,09 | 0,301 | 0,023 |
|  | s17 | 3,74 | 1,00 | 3,74 | 3,63 | 0,063 | *0,073* |
|  | s18 | 9,61 | 1,00 | 9,61 | 10,04 | **0,003** | **0,180** |
|  | s19 | 10,16 | 1,00 | 10,16 | 9,45 | **0,004** | **0,170** |
|  | s20 | 5,19 | 1,00 | 5,19 | 4,96 | **0,031** | *0,096* |
|  | s21 | 5,40 | 1,00 | 5,40 | 8,06 | **0,007** | *0,139* |
|  | s22 | 5,91 | 1,00 | 5,91 | 6,63 | **0,013** | *0,131* |
|  | s23 | 3,02 | 1,00 | 3,02 | 3,91 | 0,054 | *0,080* |
|  | s24 | 12,88 | 1,00 | 12,88 | 12,58 | **0,001** | **0,220** |
|  | s25 | 9,74 | 1,00 | 9,74 | 12,01 | **0,001** | **0,203** |
|  | s26 | 9,89 | 1,00 | 9,89 | 10,40 | **0,002** | **0,188** |
|  | s27 | 6,29 | 1,00 | 6,29 | 5,20 | **0,027** | *0,105* |
|  | s28 | 6,62 | 1,00 | 6,62 | 8,03 | **0,007** | **0,147** |
|  | s29 | 9,85 | 1,00 | 9,85 | 9,13 | **0,004** | **0,170** |
|  | s30 | 4,69 | 1,00 | 4,69 | 6,66 | **0,013** | *0,131* |
|  | s31 | 6,12 | 1,00 | 6,12 | 9,24 | **0,004** | **0,161** |
|  | s32 | 6,52 | 1,00 | 6,52 | 6,85 | **0,012** | *0,122* |
|  | s33 | 9,71 | 1,00 | 9,71 | 7,16 | **0,010** | *0,121* |
|  | s34 | 2,84 | 1,00 | 2,84 | 3,42 | 0,071 | *0,064* |
|  | s35 | 8,90 | 1,00 | 8,90 | 5,09 | **0,029** | *0,094* |
|  | s36 | 20,26 | 1,00 | 20,26 | 11,73 | **0,001** | **0,202** |
|  | s37 | 8,00 | 1,00 | 8,00 | 5,80 | **0,020** | *0,111* |
|  | s38 | 29,21 | 1,00 | 29,21 | 19,20 | **0,000** | **0,277** |
|  | s39 | 15,92 | 1,00 | 15,92 | 8,66 | **0,005** | **0,146** |
|  | s40 | 4,80 | 1,00 | 4,80 | 2,76 | 0,103 | 0,052 |
|  | s41 | 2,71 | 1,00 | 2,71 | 1,52 | 0,224 | 0,030 |
|  | s42 | 3,68 | 1,00 | 3,68 | 2,07 | 0,157 | 0,041 |
|  |  |  |  |  |  |  |  |
| Time | ST | 64,53 | 1,00 | 64,53 | 19,39 | **0,000** | **0,297** |
|  | LD | 64,24 | 1,00 | 64,24 | 20,09 | **0,000** | **0,279** |
|  | AD | 81,75 | 1,00 | 81,75 | 27,56 | **0,000** | **0,351** |
|  | TR | 30,08 | 1,00 | 30,08 | 6,96 | **0,011** | *0,132* |
|  | BI | 32,05 | 1,00 | 32,05 | 19,38 | **0,000** | **0,285** |
|  | s1 | 41,78 | 1,00 | 41,78 | 26,06 | **0,000** | **0,347** |
|  | s2 | 17,25 | 1,00 | 17,25 | 13,39 | **0,001** | **0,209** |
|  | s3 | 14,03 | 1,00 | 14,03 | 10,66 | **0,002** | **0,179** |
|  | s4 | 30,09 | 1,00 | 30,09 | 24,25 | **0,000** | **0,302** |
|  | s5 | 21,65 | 1,00 | 21,65 | 16,78 | **0,000** | **0,231** |
|  | s6 | 27,86 | 1,00 | 27,86 | 16,98 | **0,000** | **0,246** |
|  | s7 | 32,05 | 1,00 | 32,05 | 29,33 | **0,000** | **0,319** |
|  | s8 | 35,39 | 1,00 | 35,39 | 35,67 | **0,000** | **0,400** |
|  | s9 | 4,38 | 1,00 | 4,38 | 4,38 | **0,042** | *0,089* |
|  | s10 | 1,77 | 1,00 | 1,77 | 2,17 | 0,148 | 0,045 |
|  | s11 | 0,25 | 1,00 | 0,25 | 0,26 | 0,616 | 0,005 |
|  | s12 | 4,08 | 1,00 | 4,08 | 5,66 | **0,022** | *0,103* |
|  | s13 | 8,13 | 1,00 | 8,13 | 7,13 | **0,011** | *0,124* |
|  | s14 | 11,07 | 1,00 | 11,07 | 10,21 | **0,003** | **0,184** |
|  | s15 | 8,08 | 1,00 | 8,08 | 7,84 | **0,008** | **0,146** |
|  | s16 | 2,58 | 1,00 | 2,58 | 2,07 | 0,158 | 0,044 |
|  | s17 | 2,22 | 1,00 | 2,22 | 2,15 | 0,149 | 0,043 |
|  | s18 | 1,59 | 1,00 | 1,59 | 1,67 | 0,204 | 0,030 |
|  | s19 | 2,38 | 1,00 | 2,38 | 2,21 | 0,144 | 0,040 |
|  | s20 | 2,71 | 1,00 | 2,71 | 2,59 | 0,115 | 0,050 |
|  | s21 | 3,84 | 1,00 | 3,84 | 5,73 | **0,021** | *0,099* |
|  | s22 | 0,01 | 1,00 | 0,01 | 0,01 | 0,916 | 0,000 |
|  | s23 | 0,61 | 1,00 | 0,61 | 0,80 | 0,377 | 0,016 |
|  | s24 | 0,70 | 1,00 | 0,70 | 0,68 | 0,413 | 0,012 |
|  | s25 | 2,58 | 1,00 | 2,58 | 3,18 | 0,081 | 0,054 |
|  | s26 | 0,81 | 1,00 | 0,81 | 0,85 | 0,361 | 0,015 |
|  | s27 | 0,43 | 1,00 | 0,43 | 0,36 | 0,552 | 0,007 |
|  | s28 | 2,24 | 1,00 | 2,24 | 2,72 | 0,106 | 0,050 |
|  | s29 | 0,49 | 1,00 | 0,49 | 0,45 | 0,506 | 0,008 |
|  | s30 | 0,06 | 1,00 | 0,06 | 0,08 | 0,779 | 0,002 |
|  | s31 | 2,80 | 1,00 | 2,80 | 4,22 | **0,046** | *0,073* |
|  | s32 | 5,11 | 1,00 | 5,11 | 5,36 | **0,025** | *0,095* |
|  | s33 | 10,66 | 1,00 | 10,66 | 7,87 | **0,007** | *0,133* |
|  | s34 | 5,20 | 1,00 | 5,20 | 6,27 | **0,016** | *0,117* |
|  | s35 | 9,28 | 1,00 | 9,28 | 5,31 | **0,026** | *0,098* |
|  | s36 | 4,03 | 1,00 | 4,03 | 2,33 | 0,134 | 0,040 |
|  | s37 | 3,36 | 1,00 | 3,36 | 2,44 | 0,126 | 0,047 |
|  | s38 | 9,21 | 1,00 | 9,21 | 6,05 | **0,018** | *0,087* |
|  | s39 | 12,58 | 1,00 | 12,58 | 6,85 | **0,012** | *0,115* |
|  | s40 | 10,75 | 1,00 | 10,75 | 6,20 | **0,017** | *0,117* |
|  | s41 | 8,17 | 1,00 | 8,17 | 4,58 | **0,038** | *0,091* |
|  | s42 | 7,77 | 1,00 | 7,77 | 4,37 | **0,042** | *0,087* |
|  |  |  |  |  |  |  |  |
| Group | ST | 103,63 | 1,00 | 103,63 | 8,07 | **0,007** | 0,018 |
|  | LD | 16,04 | 1,00 | 16,04 | 1,15 | 0,289 | 0,003 |
|  | AD | 21,82 | 1,00 | 21,82 | 1,59 | 0,214 | 0,005 |
|  | TR | 100,63 | 1,00 | 100,63 | 5,63 | **0,022** | 0,027 |
|  | BI | 12,92 | 1,00 | 12,92 | 1,22 | 0,275 | 0,004 |
|  | s1 | 26,71 | 1,00 | 26,71 | 6,97 | **0,011** | 0,006 |
|  | s2 | 8,68 | 1,00 | 8,68 | 1,57 | 0,217 | 0,002 |
|  | s3 | 12,30 | 1,00 | 12,30 | 2,87 | 0,097 | 0,003 |
|  | s4 | 16,24 | 1,00 | 16,24 | 4,98 | **0,031** | 0,004 |
|  | s5 | 6,45 | 1,00 | 6,45 | 1,58 | 0,216 | 0,001 |
|  | s6 | 2,29 | 1,00 | 2,29 | 0,75 | 0,391 | 0,001 |
|  | s7 | 1,78 | 1,00 | 1,78 | 0,70 | 0,406 | 0,001 |
|  | s8 | 4,26 | 1,00 | 4,26 | 1,57 | 0,217 | 0,001 |
|  | s9 | 0,33 | 1,00 | 0,33 | 0,11 | 0,746 | 0,000 |
|  | s10 | 2,93 | 1,00 | 2,93 | 1,04 | 0,313 | 0,001 |
|  | s11 | 0,00 | 1,00 | 0,00 | 0,00 | 0,974 | 0,000 |
|  | s12 | 4,62 | 1,00 | 4,62 | 1,07 | 0,307 | 0,002 |
|  | s13 | 0,00 | 1,00 | 0,00 | 0,00 | 0,999 | 0,000 |
|  | s14 | 0,59 | 1,00 | 0,59 | 0,14 | 0,714 | 0,000 |
|  | s15 | 16,19 | 1,00 | 16,19 | 1,81 | 0,185 | 0,004 |
|  | s16 | 70,89 | 1,00 | 70,89 | 7,21 | **0,010** | 0,017 |
|  | s17 | 25,34 | 1,00 | 25,34 | 4,33 | **0,043** | 0,005 |
|  | s18 | 4,10 | 1,00 | 4,10 | 0,75 | 0,391 | 0,001 |
|  | s19 | 2,93 | 1,00 | 2,93 | 0,38 | 0,538 | 0,001 |
|  | s20 | 9,24 | 1,00 | 9,24 | 1,46 | 0,234 | 0,003 |
|  | s21 | 2,50 | 1,00 | 2,50 | 0,86 | 0,360 | 0,001 |
|  | s22 | 14,93 | 1,00 | 14,93 | 2,41 | 0,128 | 0,004 |
|  | s23 | 49,53 | 1,00 | 49,53 | 5,47 | **0,024** | 0,013 |
|  | s24 | 9,73 | 1,00 | 9,73 | 1,38 | 0,246 | 0,003 |
|  | s25 | 1,48 | 1,00 | 1,48 | 0,18 | 0,670 | 0,000 |
|  | s26 | 0,46 | 1,00 | 0,46 | 0,05 | 0,831 | 0,000 |
|  | s27 | 22,95 | 1,00 | 22,95 | 2,88 | 0,097 | 0,007 |
|  | s28 | 16,46 | 1,00 | 16,46 | 2,85 | 0,099 | 0,005 |
|  | s29 | 30,22 | 1,00 | 30,22 | 4,42 | **0,041** | 0,007 |
|  | s30 | 54,97 | 1,00 | 54,97 | 10,77 | **0,002** | 0,022 |
|  | s31 | 9,35 | 1,00 | 9,35 | 2,40 | 0,128 | 0,004 |
|  | s32 | 0,56 | 1,00 | 0,56 | 0,08 | 0,773 | 0,000 |
|  | s33 | 13,35 | 1,00 | 13,35 | 1,64 | 0,207 | 0,005 |
|  | s34 | 14,51 | 1,00 | 14,51 | 1,76 | 0,192 | 0,004 |
|  | s35 | 6,03 | 1,00 | 6,03 | 0,76 | 0,388 | 0,002 |
|  | s36 | 22,00 | 1,00 | 22,00 | 4,18 | **0,047** | 0,005 |
|  | s37 | 31,13 | 1,00 | 31,13 | 3,44 | 0,070 | 0,007 |
|  | s38 | 13,82 | 1,00 | 13,82 | 2,20 | 0,145 | 0,003 |
|  | s39 | 23,39 | 1,00 | 23,39 | 6,74 | **0,013** | 0,005 |
|  | s40 | 25,87 | 1,00 | 25,87 | 6,24 | **0,016** | 0,006 |
|  | s41 | 24,86 | 1,00 | 24,86 | 5,98 | **0,019** | 0,006 |
|  | s42 | 8,69 | 1,00 | 8,69 | 1,24 | 0,272 | 0,002 |

Bold pValues are statistically significant. Italic ƞ² are medium effect size. Bold ƞ² are large effect size. BI: biceps brachii; TR: triceps brachii; AD: anterior deltoid; LD: lateral deltoid; ST: superior trapezius. s1 to s42 are for the EMG signal following the map presented in the main manuscript from c1 to c7.

Table S2: Two-way ANOVA results for EMG activation variability of the Chord task.

|  |  | SumSq | DF | MeanSq | F | pValue | Ƞ² |
| --- | --- | --- | --- | --- | --- | --- | --- |
| Interaction | ST | 3,70 | 1,00 | 3,70 | 2,69 | 0,108 | 0,054 |
|  | LD | 1,78 | 1,00 | 1,78 | 1,69 | 0,200 | 0,036 |
|  | AD | 2,00 | 1,00 | 2,00 | 1,73 | 0,195 | 0,037 |
|  | TR | 0,66 | 1,00 | 0,66 | 0,74 | 0,394 | 0,015 |
|  | BI | 0,07 | 1,00 | 0,07 | 0,08 | 0,783 | 0,001 |
|  | s1 | 9,34 | 1,00 | 9,34 | 6,03 | **0,018** | *0,118* |
|  | s2 | 2,43 | 1,00 | 2,43 | 0,97 | 0,330 | 0,021 |
|  | s3 | 1,14 | 1,00 | 1,14 | 0,47 | 0,497 | 0,010 |
|  | s4 | 0,52 | 1,00 | 0,52 | 0,27 | 0,605 | 0,006 |
|  | s5 | 4,38 | 1,00 | 4,38 | 3,14 | 0,083 | *0,063* |
|  | s6 | 0,00 | 1,00 | 0,00 | 0,00 | 0,988 | 0,000 |
|  | s7 | 2,50 | 1,00 | 2,50 | 1,43 | 0,238 | 0,028 |
|  | s8 | 6,66 | 1,00 | 6,66 | 3,33 | 0,074 | *0,068* |
|  | s9 | 7,37 | 1,00 | 7,37 | 2,53 | 0,119 | 0,053 |
|  | s10 | 5,77 | 1,00 | 5,77 | 2,29 | 0,138 | 0,046 |
|  | s11 | 2,67 | 1,00 | 2,67 | 1,17 | 0,285 | 0,025 |
|  | s12 | 9,43 | 1,00 | 9,43 | 4,29 | **0,044** | *0,084* |
|  | s13 | 14,60 | 1,00 | 14,60 | 5,70 | **0,021** | *0,111* |
|  | s14 | 12,73 | 1,00 | 12,73 | 6,78 | **0,012** | *0,131* |
|  | s15 | 11,67 | 1,00 | 11,67 | 5,86 | **0,020** | *0,115* |
|  | s16 | 8,67 | 1,00 | 8,67 | 4,55 | **0,038** | *0,088* |
|  | s17 | 3,14 | 1,00 | 3,14 | 2,28 | 0,138 | 0,044 |
|  | s18 | 5,56 | 1,00 | 5,56 | 4,46 | **0,040** | *0,083* |
|  | s19 | 3,01 | 1,00 | 3,01 | 2,26 | 0,140 | 0,047 |
|  | s20 | 9,99 | 1,00 | 9,99 | 7,46 | **0,009** | *0,139* |
|  | s21 | 4,95 | 1,00 | 4,95 | 3,64 | 0,063 | *0,074* |
|  | s22 | 5,50 | 1,00 | 5,50 | 2,47 | 0,123 | 0,052 |
|  | s23 | 3,12 | 1,00 | 3,12 | 1,38 | 0,246 | 0,028 |
|  | s24 | 7,80 | 1,00 | 7,80 | 6,51 | **0,014** | *0,119* |
|  | s25 | 1,71 | 1,00 | 1,71 | 1,33 | 0,254 | 0,025 |
|  | s26 | 1,95 | 1,00 | 1,95 | 1,11 | 0,297 | 0,023 |
|  | s27 | 9,06 | 1,00 | 9,06 | 8,01 | **0,007** | **0,150** |
|  | s28 | 5,68 | 1,00 | 5,68 | 5,06 | **0,029** | *0,096* |
|  | s29 | 2,28 | 1,00 | 2,28 | 1,23 | 0,273 | 0,026 |
|  | s30 | 1,71 | 1,00 | 1,71 | 0,83 | 0,367 | 0,018 |
|  | s31 | 3,02 | 1,00 | 3,02 | 1,75 | 0,193 | 0,034 |
|  | s32 | 5,97 | 1,00 | 5,97 | 5,90 | **0,019** | **0,100** |
|  | s33 | 1,09 | 1,00 | 1,09 | 0,79 | 0,380 | 0,016 |
|  | s34 | 2,29 | 1,00 | 2,29 | 1,67 | 0,203 | 0,033 |
|  | s35 | 1,92 | 1,00 | 1,92 | 1,54 | 0,221 | 0,031 |
|  | s36 | 0,76 | 1,00 | 0,76 | 1,01 | 0,320 | 0,020 |
|  | s37 | 0,58 | 1,00 | 0,58 | 0,60 | 0,443 | 0,013 |
|  | s38 | 0,29 | 1,00 | 0,29 | 0,17 | 0,684 | 0,003 |
|  | s39 | 0,69 | 1,00 | 0,69 | 0,46 | 0,500 | 0,009 |
|  | s40 | 0,89 | 1,00 | 0,89 | 0,46 | 0,499 | 0,009 |
|  | s41 | 0,28 | 1,00 | 0,28 | 0,27 | 0,607 | 0,005 |
|  | s42 | 0,69 | 1,00 | 0,69 | 0,65 | 0,424 | 0,012 |
|  |  |  |  |  |  |  |  |
| Time | ST | 2,57 | 1,00 | 2,57 | 1,87 | 0,179 | 0,038 |
|  | LD | 0,00 | 1,00 | 0,00 | 0,00 | 0,954 | 0,000 |
|  | AD | 0,09 | 1,00 | 0,09 | 0,08 | 0,783 | 0,002 |
|  | TR | 2,99 | 1,00 | 2,99 | 3,38 | 0,073 | *0,069* |
|  | BI | 8,76 | 1,00 | 8,76 | 9,11 | **0,004** | **0,168** |
|  | s1 | 0,01 | 1,00 | 0,01 | 0,01 | 0,930 | 0,000 |
|  | s2 | 0,03 | 1,00 | 0,03 | 0,01 | 0,910 | 0,000 |
|  | s3 | 1,04 | 1,00 | 1,04 | 0,43 | 0,516 | 0,009 |
|  | s4 | 3,13 | 1,00 | 3,13 | 1,62 | 0,210 | 0,035 |
|  | s5 | 2,32 | 1,00 | 2,32 | 1,67 | 0,203 | 0,033 |
|  | s6 | 1,39 | 1,00 | 1,39 | 0,69 | 0,411 | 0,015 |
|  | s7 | 8,65 | 1,00 | 8,65 | 4,95 | **0,031** | *0,096* |
|  | s8 | 1,01 | 1,00 | 1,01 | 0,51 | 0,480 | 0,010 |
|  | s9 | 0,04 | 1,00 | 0,04 | 0,01 | 0,905 | 0,000 |
|  | s10 | 5,74 | 1,00 | 5,74 | 2,27 | 0,139 | 0,046 |
|  | s11 | 0,46 | 1,00 | 0,46 | 0,20 | 0,654 | 0,004 |
|  | s12 | 4,63 | 1,00 | 4,63 | 2,11 | 0,153 | 0,041 |
|  | s13 | 2,07 | 1,00 | 2,07 | 0,81 | 0,374 | 0,016 |
|  | s14 | 0,05 | 1,00 | 0,05 | 0,03 | 0,866 | 0,001 |
|  | s15 | 0,19 | 1,00 | 0,19 | 0,10 | 0,758 | 0,002 |
|  | s16 | 3,60 | 1,00 | 3,60 | 1,89 | 0,176 | 0,037 |
|  | s17 | 6,07 | 1,00 | 6,07 | 4,42 | **0,041** | *0,086* |
|  | s18 | 5,60 | 1,00 | 5,60 | 4,48 | **0,040** | *0,083* |
|  | s19 | 0,75 | 1,00 | 0,75 | 0,56 | 0,458 | 0,012 |
|  | s20 | 1,70 | 1,00 | 1,70 | 1,27 | 0,266 | 0,024 |
|  | s21 | 1,00 | 1,00 | 1,00 | 0,73 | 0,397 | 0,015 |
|  | s22 | 0,72 | 1,00 | 0,72 | 0,32 | 0,572 | 0,007 |
|  | s23 | 5,32 | 1,00 | 5,32 | 2,35 | 0,132 | 0,048 |
|  | s24 | 4,01 | 1,00 | 4,01 | 3,35 | 0,074 | *0,061* |
|  | s25 | 8,04 | 1,00 | 8,04 | 6,29 | **0,016** | **0,119** |
|  | s26 | 3,91 | 1,00 | 3,91 | 2,24 | 0,141 | 0,046 |
|  | s27 | 0,50 | 1,00 | 0,50 | 0,44 | 0,512 | 0,008 |
|  | s28 | 3,10 | 1,00 | 3,10 | 2,76 | 0,104 | 0,052 |
|  | s29 | 3,47 | 1,00 | 3,47 | 1,88 | 0,177 | 0,039 |
|  | s30 | 0,00 | 1,00 | 0,00 | 0,00 | 0,970 | 0,000 |
|  | s31 | 9,19 | 1,00 | 9,19 | 5,32 | **0,026** | *0,102* |
|  | s32 | 8,51 | 1,00 | 8,51 | 8,41 | **0,006** | **0,142** |
|  | s33 | 4,46 | 1,00 | 4,46 | 3,22 | 0,080 | *0,066* |
|  | s34 | 4,68 | 1,00 | 4,68 | 3,41 | 0,071 | *0,068* |
|  | s35 | 3,45 | 1,00 | 3,45 | 2,76 | 0,103 | 0,056 |
|  | s36 | 2,93 | 1,00 | 2,93 | 3,92 | 0,054 | *0,079* |
|  | s37 | 0,37 | 1,00 | 0,37 | 0,39 | 0,538 | 0,008 |
|  | s38 | 8,24 | 1,00 | 8,24 | 4,73 | **0,035** | *0,095* |
|  | s39 | 7,79 | 1,00 | 7,79 | 5,20 | **0,027** | *0,103* |
|  | s40 | 9,07 | 1,00 | 9,07 | 4,73 | **0,035** | *0,094* |
|  | s41 | 7,61 | 1,00 | 7,61 | 7,24 | **0,010** | *0,138* |
|  | s42 | 9,10 | 1,00 | 9,10 | 8,65 | **0,005** | **0,159** |
|  |  |  |  |  |  |  |  |
| Group | ST | 0,95 | 1,00 | 0,95 | 0,15 | 0,698 | 0,000 |
|  | LD | 0,22 | 1,00 | 0,22 | 0,04 | 0,844 | 0,000 |
|  | AD | 2,16 | 1,00 | 2,16 | 0,39 | 0,537 | 0,001 |
|  | TR | 2,76 | 1,00 | 2,76 | 0,95 | 0,334 | 0,003 |
|  | BI | 0,88 | 1,00 | 0,88 | 0,17 | 0,682 | 0,001 |
|  | s1 | 0,95 | 1,00 | 0,95 | 0,25 | 0,618 | 0,000 |
|  | s2 | 15,12 | 1,00 | 15,12 | 3,31 | 0,076 | 0,003 |
|  | s3 | 7,64 | 1,00 | 7,64 | 2,33 | 0,134 | 0,002 |
|  | s4 | 15,47 | 1,00 | 15,47 | 3,34 | 0,074 | 0,003 |
|  | s5 | 17,75 | 1,00 | 17,75 | 3,00 | 0,090 | 0,004 |
|  | s6 | 16,92 | 1,00 | 16,92 | 3,34 | 0,074 | 0,003 |
|  | s7 | 12,60 | 1,00 | 12,60 | 1,69 | 0,201 | 0,003 |
|  | s8 | 2,85 | 1,00 | 2,85 | 0,43 | 0,514 | 0,001 |
|  | s9 | 7,54 | 1,00 | 7,54 | 1,12 | 0,295 | 0,001 |
|  | s10 | 13,72 | 1,00 | 13,72 | 2,01 | 0,163 | 0,003 |
|  | s11 | 29,24 | 1,00 | 29,24 | 5,71 | **0,021** | 0,006 |
|  | s12 | 9,32 | 1,00 | 9,32 | 1,62 | 0,210 | 0,002 |
|  | s13 | 9,63 | 1,00 | 9,63 | 2,48 | 0,122 | 0,002 |
|  | s14 | 5,40 | 1,00 | 5,40 | 0,93 | 0,339 | 0,001 |
|  | s15 | 13,51 | 1,00 | 13,51 | 1,96 | 0,168 | 0,004 |
|  | s16 | 16,00 | 1,00 | 16,00 | 2,26 | 0,140 | 0,006 |
|  | s17 | 2,84 | 1,00 | 2,84 | 0,72 | 0,399 | 0,001 |
|  | s18 | 5,50 | 1,00 | 5,50 | 1,04 | 0,313 | 0,002 |
|  | s19 | 6,09 | 1,00 | 6,09 | 1,36 | 0,250 | 0,003 |
|  | s20 | 0,09 | 1,00 | 0,09 | 0,03 | 0,868 | 0,000 |
|  | s21 | 9,21 | 1,00 | 9,21 | 2,49 | 0,122 | 0,005 |
|  | s22 | 7,78 | 1,00 | 7,78 | 1,86 | 0,179 | 0,004 |
|  | s23 | 13,13 | 1,00 | 13,13 | 2,14 | 0,150 | 0,006 |
|  | s24 | 0,99 | 1,00 | 0,99 | 0,22 | 0,640 | 0,000 |
|  | s25 | 3,28 | 1,00 | 3,28 | 0,69 | 0,410 | 0,002 |
|  | s26 | 0,70 | 1,00 | 0,70 | 0,16 | 0,687 | 0,000 |
|  | s27 | 11,58 | 1,00 | 11,58 | 2,32 | 0,134 | 0,006 |
|  | s28 | 3,51 | 1,00 | 3,51 | 0,83 | 0,368 | 0,002 |
|  | s29 | 3,25 | 1,00 | 3,25 | 0,98 | 0,327 | 0,002 |
|  | s30 | 3,99 | 1,00 | 3,99 | 0,80 | 0,375 | 0,003 |
|  | s31 | 4,73 | 1,00 | 4,73 | 0,84 | 0,363 | 0,003 |
|  | s32 | 10,60 | 1,00 | 10,60 | 3,21 | 0,080 | 0,008 |
|  | s33 | 19,09 | 1,00 | 19,09 | 4,18 | **0,047** | 0,013 |
|  | s34 | 3,37 | 1,00 | 3,37 | 0,82 | 0,369 | 0,003 |
|  | s35 | 1,55 | 1,00 | 1,55 | 0,42 | 0,521 | 0,001 |
|  | s36 | 7,39 | 1,00 | 7,39 | 2,26 | 0,140 | 0,006 |
|  | s37 | 3,45 | 1,00 | 3,45 | 0,52 | 0,474 | 0,002 |
|  | s38 | 4,25 | 1,00 | 4,25 | 0,95 | 0,334 | 0,002 |
|  | s39 | 3,14 | 1,00 | 3,14 | 0,81 | 0,373 | 0,002 |
|  | s40 | 6,30 | 1,00 | 6,30 | 2,05 | 0,159 | 0,005 |
|  | s41 | 5,46 | 1,00 | 5,46 | 1,80 | 0,187 | 0,004 |
|  | s42 | 3,36 | 1,00 | 3,36 | 0,76 | 0,388 | 0,002 |

Bold pValues are statistically significant. Italic ƞ² are medium effect size. Bold ƞ² are large effect size. BI: biceps brachii; TR: triceps brachii; AD: anterior deltoid; LD: lateral deltoid; ST: superior trapezius. s1 to s42 are for the EMG signal following the map presented in the main manuscript from c1 to c7.

Table S3: Two-way ANOVA results for acceleration variability of the Digital task.

|  | Segment | SumSq | DF | MeanSq | F | pValue | Ƞ² |
| --- | --- | --- | --- | --- | --- | --- | --- |
| Interaction | Pelvis | 0,00 | 1,00 | 0,00 | 0,39 | 0,534 | 0,006 |
|  | Thorax | 0,00 | 1,00 | 0,00 | 0,49 | 0,488 | 0,008 |
|  | Head | 0,01 | 1,00 | 0,01 | 3,57 | 0,065 | *0,067* |
|  | rScapula | 0,00 | 1,00 | 0,00 | 3,11 | 0,085 | 0,035 |
|  | rArm | 0,07 | 1,00 | 0,07 | 6,59 | **0,014** | *0,067* |
|  | rForearm | 0,01 | 1,00 | 0,01 | 0,93 | 0,340 | 0,012 |
|  | rWrist | 0,03 | 1,00 | 0,03 | 1,21 | 0,277 | 0,017 |
|  |  |  |  |  |  |  |  |
| Time | Pelvis | 0,00 | 1,00 | 0,00 | 18,28 | **0,000** | **0,292** |
|  | Thorax | 0,01 | 1,00 | 0,01 | 16,90 | **0,000** | **0,275** |
|  | Head | 0,02 | 1,00 | 0,02 | 5,56 | **0,023** | *0,105* |
|  | rScapula | 0,04 | 1,00 | 0,04 | 41,67 | **0,000** | **0,469** |
|  | rArm | 0,51 | 1,00 | 0,51 | 47,77 | **0,000** | **0,486** |
|  | rForearm | 0,51 | 1,00 | 0,51 | 33,27 | **0,000** | **0,425** |
|  | rWrist | 0,66 | 1,00 | 0,66 | 27,72 | **0,000** | **0,380** |
|  |  |  |  |  |  |  |  |
| Group | Pelvis | 0,00 | 1,00 | 0,00 | 0,39 | 0,533 | 0,002 |
|  | Thorax | 0,00 | 1,00 | 0,00 | 0,11 | 0,743 | 0,000 |
|  | Head | 0,00 | 1,00 | 0,00 | 0,11 | 0,740 | 0,000 |
|  | rScapula | 0,00 | 1,00 | 0,00 | 0,01 | 0,905 | 0,000 |
|  | rArm | 0,02 | 1,00 | 0,02 | 0,30 | 0,584 | 0,001 |
|  | rForearm | 0,00 | 1,00 | 0,00 | 0,03 | 0,866 | 0,000 |
|  | rWrist | 0,00 | 1,00 | 0,00 | 0,00 | 0,953 | 0,000 |

Bold pValues are statistically significant. Italic ƞ² are medium effect size. Bold ƞ² are large effect size.

Table S4: Two-way ANOVA results for acceleration variability of the Chord task.

|  |  | SumSq | DF | MeanSq | F | pValue | Ƞ² |
| --- | --- | --- | --- | --- | --- | --- | --- |
| Interaction | Pelvis | 0,00 | 1,00 | 0,00 | 0,31 | 0,580 | 0,007 |
|  | Thorax | 0,03 | 1,00 | 0,03 | 5,81 | **0,020** | *0,111* |
|  | Head | 0,07 | 1,00 | 0,07 | 5,93 | **0,019** | *0,116* |
|  | rScapula | 0,02 | 1,00 | 0,02 | 1,52 | 0,224 | 0,032 |
|  | rArm | 0,03 | 1,00 | 0,03 | 0,30 | 0,585 | 0,006 |
|  | rForearm | 0,00 | 1,00 | 0,00 | 0,00 | 0,949 | 0,000 |
|  | rWrist | 0,04 | 1,00 | 0,04 | 0,06 | 0,808 | 0,001 |
|  |  |  |  |  |  |  |  |
| Time | Pelvis | 0,00 | 1,00 | 0,00 | 0,44 | 0,510 | 0,010 |
|  | Thorax | 0,01 | 1,00 | 0,01 | 1,74 | 0,193 | 0,033 |
|  | Head | 0,00 | 1,00 | 0,00 | 0,03 | 0,859 | 0,001 |
|  | rScapula | 0,01 | 1,00 | 0,01 | 0,65 | 0,426 | 0,014 |
|  | rArm | 0,28 | 1,00 | 0,28 | 2,78 | 0,102 | 0,058 |
|  | rForearm | 2,20 | 1,00 | 2,20 | 9,53 | **0,003** | **0,175** |
|  | rWrist | 4,17 | 1,00 | 4,17 | 6,63 | **0,013** | *0,128* |
|  |  |  |  |  |  |  |  |
| Group | Pelvis | 0,02 | 1,00 | 0,02 | 1,80 | 0,186 | 0,007 |
|  | Thorax | 0,14 | 1,00 | 0,14 | 2,61 | 0,113 | 0,006 |
|  | Head | 0,01 | 1,00 | 0,01 | 0,16 | 0,690 | 0,000 |
|  | rScapula | 0,01 | 1,00 | 0,01 | 0,30 | 0,584 | 0,000 |
|  | rArm | 0,02 | 1,00 | 0,02 | 0,07 | 0,793 | 0,000 |
|  | rForearm | 0,11 | 1,00 | 0,11 | 0,14 | 0,714 | 0,000 |
|  | rWrist | 0,21 | 1,00 | 0,21 | 0,15 | 0,698 | 0,000 |

Bold pValues are statistically significant. Italic ƞ² are medium effect size. Bold ƞ² are large effect size.

Table S5: Two-way ANOVA results for angle variability of the Digital task.

|  |  | SumSq | DF | MeanSq | F | pValue | Ƞ² |
| --- | --- | --- | --- | --- | --- | --- | --- |
| Interaction | Pelvis tilt | 0,15 | 1,00 | 0,15 | 0,40 | 0,529 | 0,008 |
|  | Pelvis elevation | 0,00 | 1,00 | 0,00 | 0,01 | 0,930 | 0,000 |
|  | Pelvis rotation | 0,00 | 1,00 | 0,00 | 0,06 | 0,815 | 0,001 |
|  | Thorax flexion | 1,84 | 1,00 | 1,84 | 2,02 | 0,162 | 0,037 |
|  | Thorax lat.flex. | 0,86 | 1,00 | 0,86 | 3,83 | 0,057 | *0,077* |
|  | Thorax rotation | 0,01 | 1,00 | 0,01 | 0,08 | 0,781 | 0,001 |
|  | Neck flexion | 10,77 | 1,00 | 10,77 | 0,94 | 0,337 | 0,019 |
|  | Neck lat.flex. | 7,93 | 1,00 | 7,93 | 1,10 | 0,300 | 0,022 |
|  | Neck rotation | 17,42 | 1,00 | 17,42 | 1,53 | 0,223 | 0,032 |
|  | rScapula tilt | 0,16 | 1,00 | 0,16 | 0,69 | 0,410 | 0,015 |
|  | rScapula elevation | 0,13 | 1,00 | 0,13 | 1,04 | 0,313 | 0,018 |
|  | rScapula rotation | 0,33 | 1,00 | 0,33 | 2,17 | 0,148 | 0,043 |
|  | rShoulder flexion | 0,76 | 1,00 | 0,76 | 1,10 | 0,300 | 0,020 |
|  | rShoulder abduction | 0,02 | 1,00 | 0,02 | 0,02 | 0,882 | 0,000 |
|  | rShoulder rotation | 0,02 | 1,00 | 0,02 | 0,01 | 0,904 | 0,000 |
|  | rElbow flexion | 0,11 | 1,00 | 0,11 | 0,07 | 0,798 | 0,001 |
|  | rElbow pronation | 0,28 | 1,00 | 0,28 | 2,29 | 0,137 | 0,038 |
|  | rWrist flexion | 0,20 | 1,00 | 0,20 | 0,59 | 0,447 | 0,013 |
|  | rWrist abduction | 0,48 | 1,00 | 0,48 | 2,02 | 0,162 | 0,042 |
|  |  |  |  |  |  |  |  |
| Time | Pelvis tilt | 2,79 | 1,00 | 2,79 | 7,73 | **0,008** | **0,148** |
|  | Pelvis elevation | 0,88 | 1,00 | 0,88 | 8,89 | **0,005** | **0,168** |
|  | Pelvis rotation | 0,46 | 1,00 | 0,46 | 8,75 | **0,005** | **0,166** |
|  | Thorax flexion | 7,98 | 1,00 | 7,98 | 8,75 | **0,005** | **0,160** |
|  | Thorax lat.flex. | 0,49 | 1,00 | 0,49 | 2,21 | 0,145 | 0,044 |
|  | Thorax rotation | 4,13 | 1,00 | 4,13 | 23,52 | **0,000** | **0,348** |
|  | Neck flexion | 49,72 | 1,00 | 49,72 | 4,35 | **0,043** | *0,088* |
|  | Neck lat.flex. | 30,61 | 1,00 | 30,61 | 4,24 | **0,045** | *0,086* |
|  | Neck rotation | 27,84 | 1,00 | 27,84 | 2,44 | 0,126 | 0,051 |
|  | rScapula tilt | 0,46 | 1,00 | 0,46 | 1,97 | 0,167 | 0,042 |
|  | rScapula elevation | 1,55 | 1,00 | 1,55 | 12,27 | **0,001** | **0,214** |
|  | rScapula rotation | 0,66 | 1,00 | 0,66 | 4,29 | **0,044** | 0,085 |
|  | rShoulder flexion | 6,72 | 1,00 | 6,72 | 9,81 | **0,003** | **0,179** |
|  | rShoulder abduction | 1,96 | 1,00 | 1,96 | 2,35 | 0,133 | 0,051 |
|  | rShoulder rotation | 2,86 | 1,00 | 2,86 | 2,43 | 0,126 | 0,052 |
|  | rElbow flexion | 8,92 | 1,00 | 8,92 | 5,40 | **0,025** | *0,109* |
|  | rElbow pronation | 1,77 | 1,00 | 1,77 | 14,68 | **0,000** | **0,241** |
|  | rWrist flexion | 0,46 | 1,00 | 0,46 | 1,40 | 0,244 | 0,030 |
|  | rWrist abduction | 0,55 | 1,00 | 0,55 | 2,31 | 0,135 | 0,048 |
|  |  |  |  |  |  |  |  |
| Group | Pelvis tilt | 0,01 | 1,00 | 0,01 | 0,01 | 0,912 | 0,000 |
|  | Pelvis elevation | 0,03 | 1,00 | 0,03 | 0,18 | 0,674 | 0,002 |
|  | Pelvis rotation | 0,03 | 1,00 | 0,03 | 0,36 | 0,554 | 0,003 |
|  | Thorax flexion | 0,03 | 1,00 | 0,03 | 0,02 | 0,885 | 0,000 |
|  | Thorax lat.flex. | 0,02 | 1,00 | 0,02 | 0,04 | 0,838 | 0,000 |
|  | Thorax rotation | 0,09 | 1,00 | 0,09 | 0,18 | 0,677 | 0,001 |
|  | Neck flexion | 7,93 | 1,00 | 7,93 | 0,25 | 0,619 | 0,002 |
|  | Neck lat.flex. | 5,17 | 1,00 | 5,17 | 0,25 | 0,616 | 0,003 |
|  | Neck rotation | 0,14 | 1,00 | 0,14 | 0,01 | 0,932 | 0,000 |
|  | rScapula tilt | 0,17 | 1,00 | 0,17 | 0,39 | 0,536 | 0,003 |
|  | rScapula elevation | 0,00 | 1,00 | 0,00 | 0,01 | 0,917 | 0,000 |
|  | rScapula rotation | 0,02 | 1,00 | 0,02 | 0,05 | 0,831 | 0,000 |
|  | rShoulder flexion | 0,14 | 1,00 | 0,14 | 0,07 | 0,794 | 0,000 |
|  | rShoulder abduction | 0,35 | 1,00 | 0,35 | 0,21 | 0,648 | 0,001 |
|  | rShoulder rotation | 3,34 | 1,00 | 3,34 | 0,83 | 0,368 | 0,005 |
|  | rElbow flexion | 1,45 | 1,00 | 1,45 | 0,47 | 0,496 | 0,004 |
|  | rElbow pronation | 0,07 | 1,00 | 0,07 | 0,15 | 0,704 | 0,000 |
|  | rWrist flexion | 1,69 | 1,00 | 1,69 | 1,53 | 0,223 | 0,003 |
|  | rWrist abduction | 0,05 | 1,00 | 0,05 | 0,09 | 0,767 | 0,000 |

Bold pValues are statistically significant. Italic ƞ² are medium effect size. Bold ƞ² are large effect size.

Table S6: Two-way ANOVA results for angle variability of the Chord task.

|  |  | SumSq | DF | MeanSq | F | pValue | Ƞ² |
| --- | --- | --- | --- | --- | --- | --- | --- |
| Interaction | Pelvis tilt | 0,01 | 1,00 | 0,01 | 0,04 | 0,840 | 0,001 |
|  | Pelvis elevation | 0,04 | 1,00 | 0,04 | 0,14 | 0,707 | 0,003 |
|  | Pelvis rotation | 0,07 | 1,00 | 0,07 | 1,13 | 0,293 | 0,024 |
|  | Thorax flexion | 1,58 | 1,00 | 1,58 | 3,22 | 0,079 | *0,061* |
|  | Thorax lat.flex. | 0,14 | 1,00 | 0,14 | 0,52 | 0,474 | 0,011 |
|  | Thorax rotation | 0,40 | 1,00 | 0,40 | 2,96 | 0,092 | 0,059 |
|  | Neck flexion | 3,50 | 1,00 | 3,50 | 0,54 | 0,465 | 0,012 |
|  | Neck lat.flex. | 0,06 | 1,00 | 0,06 | 0,06 | 0,811 | 0,001 |
|  | Neck rotation | 5,31 | 1,00 | 5,31 | 2,75 | 0,104 | 0,057 |
|  | rScapula tilt | 0,05 | 1,00 | 0,05 | 0,37 | 0,546 | 0,008 |
|  | rScapula elevation | 0,01 | 1,00 | 0,01 | 0,09 | 0,762 | 0,002 |
|  | rScapula rotation | 0,06 | 1,00 | 0,06 | 0,43 | 0,515 | 0,009 |
|  | rShoulder flexion | 0,87 | 1,00 | 0,87 | 2,10 | 0,155 | 0,042 |
|  | rShoulder abduction | 0,08 | 1,00 | 0,08 | 0,14 | 0,709 | 0,003 |
|  | rShoulder rotation | 0,16 | 1,00 | 0,16 | 0,32 | 0,574 | 0,007 |
|  | rElbow flexion | 0,13 | 1,00 | 0,13 | 0,28 | 0,601 | 0,006 |
|  | rElbow pronation | 0,14 | 1,00 | 0,14 | 0,47 | 0,495 | 0,010 |
|  | rWrist flexion | 0,12 | 1,00 | 0,12 | 0,20 | 0,657 | 0,004 |
|  | rWrist abduction | 0,03 | 1,00 | 0,03 | 0,13 | 0,723 | 0,003 |
|  |  |  |  |  |  |  |  |
| Time | Pelvis tilt | 0,21 | 1,00 | 0,21 | 0,94 | 0,337 | 0,020 |
|  | Pelvis elevation | 0,00 | 1,00 | 0,00 | 0,01 | 0,916 | 0,000 |
|  | Pelvis rotation | 0,05 | 1,00 | 0,05 | 0,81 | 0,374 | 0,017 |
|  | Thorax flexion | 2,11 | 1,00 | 2,11 | 4,32 | **0,044** | *0,082* |
|  | Thorax lat.flex. | 0,34 | 1,00 | 0,34 | 1,25 | 0,269 | 0,027 |
|  | Thorax rotation | 0,33 | 1,00 | 0,33 | 2,45 | 0,124 | 0,049 |
|  | Neck flexion | 1,21 | 1,00 | 1,21 | 0,19 | 0,667 | 0,004 |
|  | Neck lat.flex. | 10,35 | 1,00 | 10,35 | 9,28 | **0,004** | **0,171** |
|  | Neck rotation | 0,85 | 1,00 | 0,85 | 0,44 | 0,510 | 0,009 |
|  | rScapula tilt | 0,00 | 1,00 | 0,00 | 0,03 | 0,872 | 0,001 |
|  | rScapula elevation | 0,31 | 1,00 | 0,31 | 2,93 | 0,094 | *0,061* |
|  | rScapula rotation | 0,21 | 1,00 | 0,21 | 1,49 | 0,228 | 0,032 |
|  | rShoulder flexion | 1,11 | 1,00 | 1,11 | 2,67 | 0,109 | 0,054 |
|  | rShoulder abduction | 0,14 | 1,00 | 0,14 | 0,26 | 0,614 | 0,006 |
|  | rShoulder rotation | 0,41 | 1,00 | 0,41 | 0,84 | 0,364 | 0,018 |
|  | rElbow flexion | 0,01 | 1,00 | 0,01 | 0,02 | 0,882 | 0,000 |
|  | rElbow pronation | 0,01 | 1,00 | 0,01 | 0,02 | 0,877 | 0,001 |
|  | rWrist flexion | 0,75 | 1,00 | 0,75 | 1,25 | 0,270 | 0,027 |
|  | rWrist abduction | 0,17 | 1,00 | 0,17 | 0,64 | 0,428 | 0,014 |
|  |  |  |  |  |  |  |  |
| Group | Pelvis tilt | 0,65 | 1,00 | 0,65 | 0,67 | 0,416 | 0,004 |
|  | Pelvis elevation | 0,00 | 1,00 | 0,00 | 0,00 | 0,987 | 0,000 |
|  | Pelvis rotation | 0,06 | 1,00 | 0,06 | 0,37 | 0,545 | 0,001 |
|  | Thorax flexion | 0,03 | 1,00 | 0,03 | 0,01 | 0,907 | 0,000 |
|  | Thorax lat.flex. | 0,07 | 1,00 | 0,07 | 0,16 | 0,692 | 0,000 |
|  | Thorax rotation | 0,21 | 1,00 | 0,21 | 0,39 | 0,534 | 0,001 |
|  | Neck flexion | 9,49 | 1,00 | 9,49 | 0,83 | 0,368 | 0,004 |
|  | Neck lat.flex. | 4,79 | 1,00 | 4,79 | 2,41 | 0,127 | 0,009 |
|  | Neck rotation | 3,06 | 1,00 | 3,06 | 1,08 | 0,305 | 0,003 |
|  | rScapula tilt | 0,02 | 1,00 | 0,02 | 0,06 | 0,811 | 0,000 |
|  | rScapula elevation | 0,25 | 1,00 | 0,25 | 0,88 | 0,352 | 0,002 |
|  | rScapula rotation | 0,01 | 1,00 | 0,01 | 0,04 | 0,837 | 0,000 |
|  | rShoulder flexion | 0,05 | 1,00 | 0,05 | 0,04 | 0,852 | 0,000 |
|  | rShoulder abduction | 0,01 | 1,00 | 0,01 | 0,01 | 0,922 | 0,000 |
|  | rShoulder rotation | 0,91 | 1,00 | 0,91 | 0,44 | 0,509 | 0,001 |
|  | rElbow flexion | 0,88 | 1,00 | 0,88 | 0,46 | 0,502 | 0,001 |
|  | rElbow pronation | 0,00 | 1,00 | 0,00 | 0,00 | 0,980 | 0,000 |
|  | rWrist flexion | 6,76 | 1,00 | 6,76 | 4,29 | **0,044** | 0,005 |
|  | rWrist abduction | 0,72 | 1,00 | 0,72 | 0,62 | 0,436 | 0,001 |

Bold pValues are statistically significant. Italic ƞ² are medium effect size. Bold ƞ² are large effect size.
